# THC-RPL: A lightweight Trust-enabled routing in RPL-based IoT networks against Sybil attack

**DOI:** 10.1371/journal.pone.0271277

**Published:** 2022-07-28

**Authors:** Danyal Arshad, Muhammad Asim, Noshina Tariq, Thar Baker, Hissam Tawfik, Dhiya Al-Jumeily OBE

**Affiliations:** 1 Department of Computer Science, National University of Computer and Emerging Sciences, Islamabad, Pakistan; 2 Department of Avionics Engineering, Air University, Islamabad, Pakistan; 3 Department of Computer Science, College of Computing and Informatics, University of Sharjah, Sharjah, UAE; 4 Department of Electrical Engineering, College of Engineering, University of Sharjah, Sharjah, UAE; 5 School of Built Environment, Engineering and Computing, Leeds Beckett University, Leeds, United Kingdom; 6 School of Computer Science and Mathematics, Liverpool John Moores University, Liverpool, United Kingdom; University College of Engineering Tindivanam, INDIA

## Abstract

The Internet of Things (IoT) and its relevant advances have attracted significant scholarly, governmental, and industrial attention in recent years. Since the IoT specifications are quite different from what the Internet can deliver today, many groundbreaking techniques, such as Mobile Ad hoc Networks (MANETs) and Wireless Sensor Networks (WSN), have gradually been integrated into IoT. The Routing Protocol for Low power and Lossy network (RPL) is the de-facto IoT routing protocol in such networks. Unfortunately, it is susceptible to numerous internal attacks. Many techniques, such as cryptography, Intrusion Detection System (IDS), and authorization have been used to counter this. The large computational overhead of these techniques limits their direct application to IoT nodes, especially due to their low power and lossy nature. Therefore, this paper proposes a Trust-based Hybrid Cooperative RPL protocol (THC-RPL) to detect malicious Sybil nodes in an RPL-based IoT network. The proposed technique is compared and evaluated with state-of-the-art and is found to outperform them. It detects more attacks while maintaining the packet loss ratio in the range of 15-25%. The average energy consumption of the nodes also remains in the ratio of 60-80 mj. There is approximately 40% more energy conservation at node level with an overall 50% increase in network lifetime. THC-RPL has 10% less message exchange and 0% storage costs.

## 1. Introduction

With the main aim of providing intelligent and omnipresent services, the Internet of Things (IoT) is a rapidly evolving network of physical objects that detects, monitors, and gathers data [1, 2]. It has impacted nearly every industry, including banking and finance, smart homes, smart healthcare, and managing and analyzing data [3, 4]. However, security is a major concern for the ever-expanding IoT applications, where most of the IoT device infrastructure does not deal with security and privacy concerns [5]. Malicious attacks, including others, are unavoidable as the number of connected devices grows. IoT-related routing protocols have been designed to enable optimal route choices. However, they have not been properly validated for reliability. The Routing Protocol for Low Power and Lossy Networks (RPL) is an IPv6-based standard routing protocol developed for IoT devices [6].

RPL supports multi-topology routing, minimal computing and message cost, and down and upward transmission. Unfortunately, It is also prone to attacks like Sybil, blackhole, and selective forwarding attacks as it does not address network-level security [7]. Internal threats like blackhole attacks and selective forwarding are readily spotted since they result in significant control overhead, high packet loss rates, and excessive network delay. In contrast, attacks such as Sybil, employing packet forging mechanisms that appear as several identities, are challenging until the RPL routing throughput suffers. In addition, heterogeneous devices from different vendors and uncontrolled environments contribute to the increasing level and complexity of security challenges. One of the major aspects to consider in IoT is routing. Routing, scalability, autonomy, data integrity and confidentiality, secure communication, and energy efficiency are major goals to address in IoT networks [8–10].

There are many techniques used to avoid security threats on a large vulnerable space of IoT networks [11–13]. Despite the various security methods deployed to secure IoT devices, such as authentication and cryptography [14]. However, IoT devices are still susceptible to attacks owing to their limited resources and dense network dispersion. Memory and CPU cycles are required for most cryptographic techniques, resulting in a decrease in overall performance [15, 16]. The whole network’s information is at stake if the attackers get their hands on the encryption keys. When a node’s behavior is not considered during routing, security attacks like Sybil may be readily executed, opening the way for the additional internal attack. Such attacks can be prevented by employing trust-based secure routing methods. These limitations in cryptographic and authentication techniques are not suitable enough to be directly used in LLNs. Besides, cryptographic techniques are used for external attacks; they become ineffective in mitigating internal attacks [8]. It is due to the reason that the nodes are already authentic and share/know the keys.

Trust-based schemes are available to overcome the above mentioned limitations to ensure security for internal attacks. Trust-Based mechanisms observe the behavior of the neighbor nodes and then decide whether it is malicious or not [17]. However, they require heavy computations at the node level resulting in a rapid energy drain of the nodes. Hence, there is a real need to develop a lightweight yet energy-efficient trust mechanism to mitigate the Sybil attack [18]. This paper proposes a lightweight and energy-efficient Trust-enabled Hybrid Cooperative RPL protocol (THC-RPL) to detect and isolate malicious Sybil nodes in RPL-based IoT networks. It provides a solution against fake new and stolen identities in mobile and static IoT networks. The lifetime of the overall network increases by offloading computations towards the resourceful nodes. This study mainly considers the issue of rapid energy depletion of nodes, focusing on improving the energy efficiency at the node level. Secondly, it aims to decrease the computation and storage cost at the node level by offloading trust-related tasks at the root node. Third, detect and isolate the Sybil attack node in the IoT-based mobile networks. The most pertinent contributions of the paper are listed below:

1. A lightweight Trust-enabled Hybrid Cooperative RPL protocol is proposed to mitigate Sybil attack.
2. Sybil threat model and security analysis is presented using mathematical analysis.
3. An energy-efficient security solution for internal attacks is presented.
4. A novel yet naive scheme to handle static, dynamic, and mobile nature of sensor nodes (leaving and joining the topology at any time) is proposed.
5. A resource-conserved scheme is presented to offload computations towards the resourceful nodes, to preserve the scarce resources of the nodes.
6. A comprehensive comparative study among proposed scheme and other similar existing schemes is done, providing the analysis of attack detected, packet loss ratio, average energy consumed at node and network levels, computation cost, communication cost, and storage cost with benchmark results.

The rest of the paper organisation is as follows: Section 2 highlights the existing problems in RPL. Section 3 details the background and context. Literature review and proposed methodology are given in Section 4 and 5, respectively. Section 6 discusses the evaluation and results. Finally, the conclusion and future work are stated in Section 7.

## 2. Problem statement

RPL is currently considered the standard LLN routing protocol. It protects against the network’s external attacks; however, it may not protect against internal attacks, such as the Sybil attack. Internal attacks disrupt a network’s routing topology resulting in the loss of resources for IoT devices. The large and complex computations in traditional security mechanisms (e.g., cryptographic and authorization) are unsuitable for LLNs. The trust-based mechanism is used for detecting internal attacks. It detects the malicious behavior of nodes based on calculated trust. Neighbor nodes calculate the trust value to distinguish between a legitimate and a malicious node. This mechanism can also face computation complexity [7]; for instance, the T-mote sky wireless sensor module used for sensing humidity, light, and the temperature has a 16-bit processor with 10 KB RAM and 48 KB flash memory used program storage with a 2AA battery backup. When running the RPL protocol on such motes, 0.0920 watts/min energy is consumed [18]. In addition, the trust measurement also requires calculations resulting in the node’s resources getting exhausted. It reduces the performance and lifespan of resource-restricted devices where a node executes all computations and shares its ‘trust’ information with its adjacent (neighboring) nodes. Since IoT devices are inherently resource-constrained, processing at the node-level results in a rapid depletion of its resources, which reduces the IoT device’s lifetime and ultimately its applicability. Therefore, a lightweight Trust-enabled Hybrid Cooperative RPL protocol is suggested to minimize Sybil attacks. In support of the claim, a mathematical estimation is also provided to illustrate the Sybil threat model and security analysis. The proposed scheme is energy-efficient against internal assaults and deals with sensor nodes’ static, dynamic, and mobile nature. It is a resource-conserving strategy that offloads computations to resourceful nodes to save the nodes’ finite resources.

## 3. Background and context

This Section details IoTs, RPL and its working, the Sybil attack and how it disrupts a network, and the trust-based security and how it maneuvers with the internal attacks.

### 3.1 Internet of things

**Fig 1:**
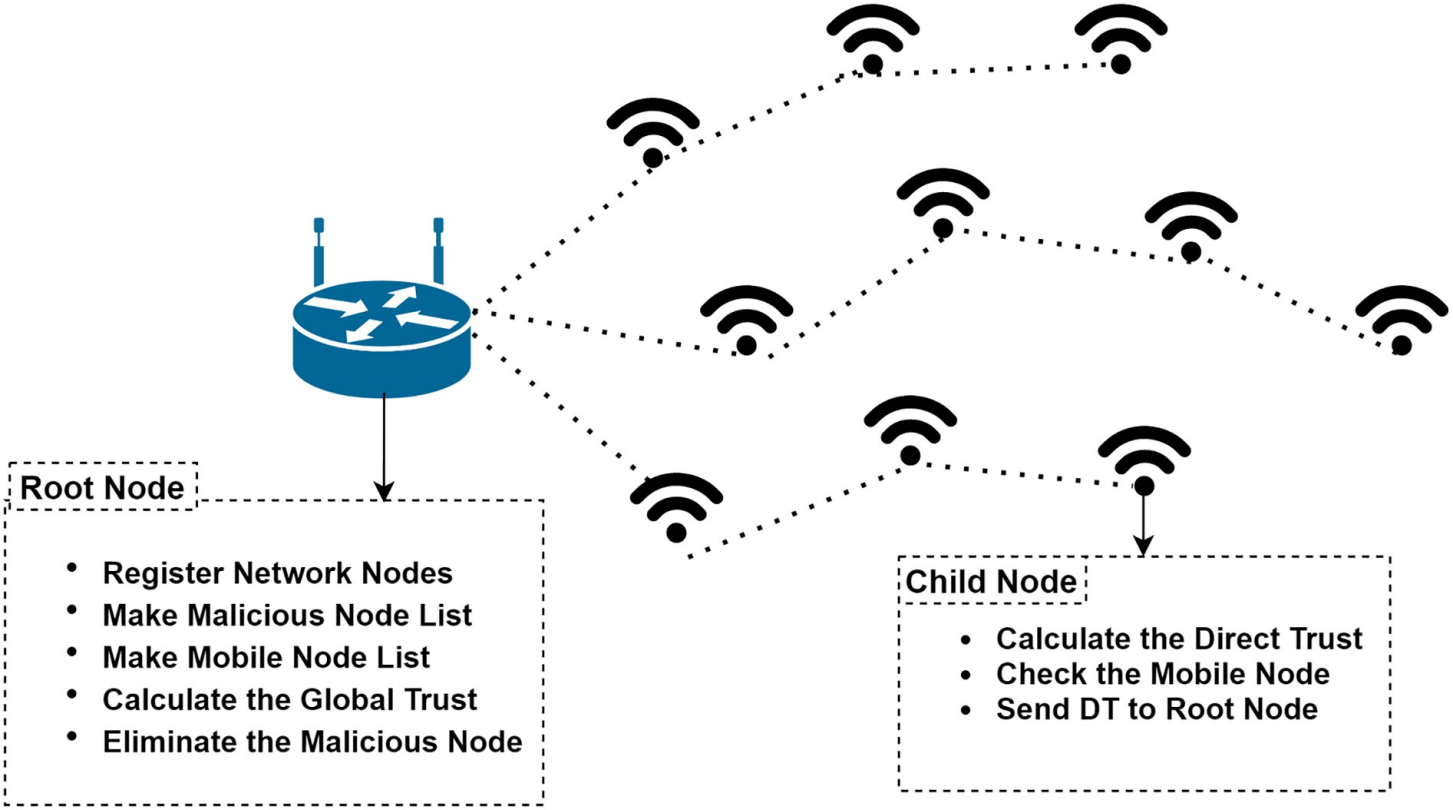
An example of IoTs.

**Fig 2:**
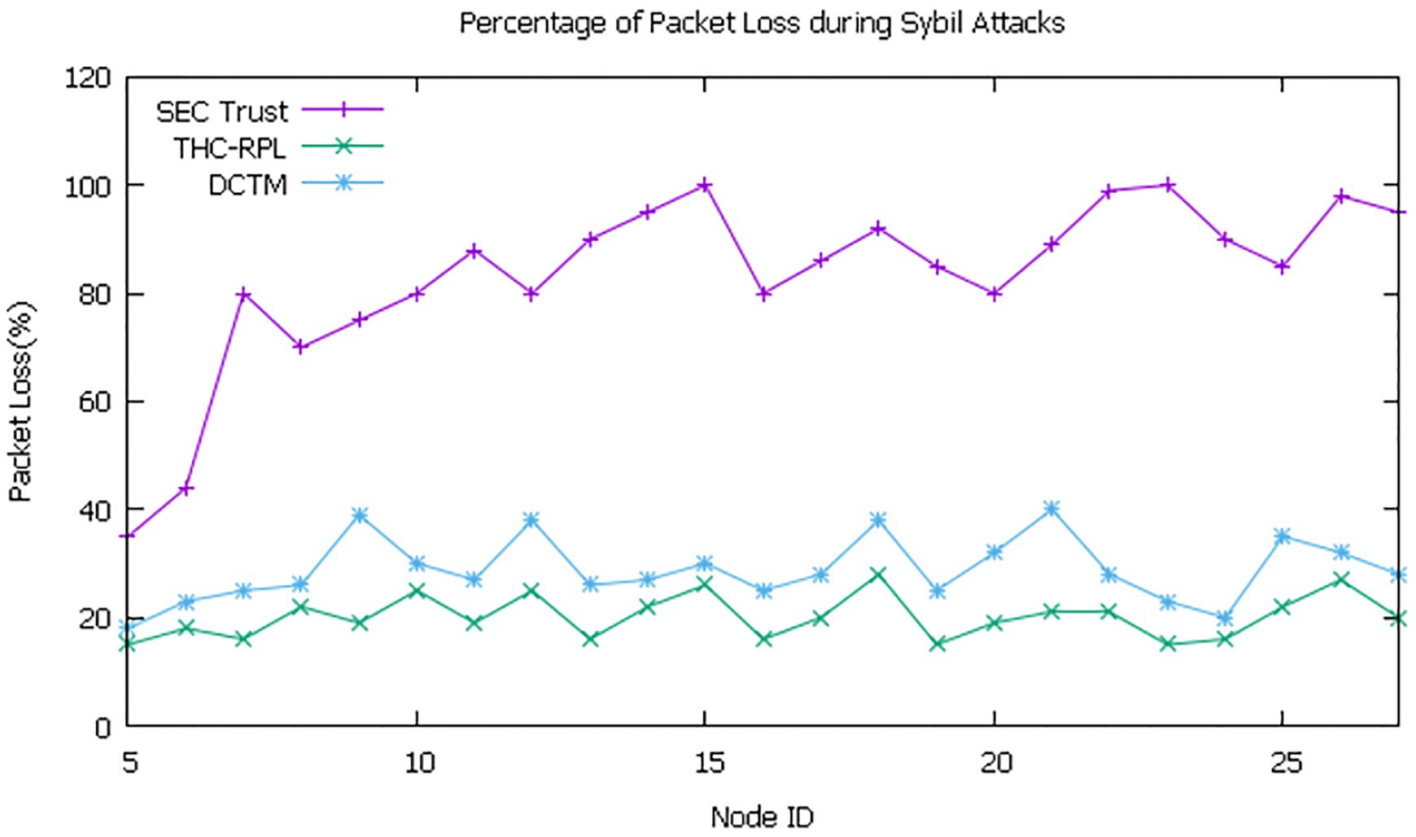
The IoT architecture.

IoT is a smart platform connecting the physical and digital worlds. Every device is becoming smarter, intelligent, and connected, as shown in Fig 1. They exchange data for analytics and decision-making. Around 50 to 100 billion devices are anticipated in 2025, according to the Cisco report [19]. These devices consist of laptops, smartphones, and other smart embedded devices. The main goal is to create smart ecosystems such as smart homes, smart cities, smart buildings, smart transport, and smart grids. The IoT architecture generally consists of five layers [20]; physical, network, middleware, application, and business layer, as shown in Fig 2. The detail of the layers is given below:

- Physical Layer: is also called a layer of sensors. It is concerned with physical objects and sensor devices. Sensors, such as bar codes reader, infrared sensors, and RFID are depended upon for IoT applications. This layer senses and collects information, such as location, temperature, humidity, and chemical changes in the air. The collected information is transmitted to the network layer for transfer and processing.
- Network Layer: is also known as a transmission layer. Using a wired or wireless medium, it transmits information safely from the application layer to the data processing unit.
- Middleware Layer: consists of IoT devices running different types of services. Devices communicate with only those devices that provide similar services. This layer provides service management and also connects to the database. Information is received from the network layer and stored in the database.
- Application Layer: provides a platform for IoT applications. These applications run on this layer, for example, smart transportation or smart home applications.
- Business Layer: manages applications and services running on IoT devices.

### 3.2 Routing protocol; RPL For LLNs

IETF proposes an independent standardized RPL routing protocol based on IPv6 for resource-restricted devices. RPL is configured for lossy connections to meet minimum routing requirements. It supports multipoint-to-point, point-to-multipoint, and point-to-point models of traffic [20]. RPL forms a tree-like topology and generates Destination Oriented Directed Acyclic Graph (DODAG) that describes the network’s topology or routing structure. DODAG is an acyclic graph that has a single root node. Every node knows about their parents; however, they do not know about their children. In RPL, every node has its preferred parent and at least one path to the root node. RPL uses four control messages to update the routing information. The first control message is the DODAG Information Object (DIO), which specifies the node’s rank concerning the root node contributing to the choice of the chosen parent. Destination Advertisement Object (DAO) is the second type of message, unicasting destination information to the parents selected. DODAG Information Solicitation (DIS) is the third type. A node uses this control message to get the DIO message from neighboring nodes. DAO Acknowledgement (DAO-ACK) is the fourth and last control message type. This control message responds to the DAO message receiver as a parent node or DODAG root node. RPL also uses an objective function, Zero Objective Function (0-OF), and Minimum Rank Hysteresis (MRHOF). 0-OF uses hop count as a routing metric, and MRHOF uses the expected transmission count metric for routing. Using a single metric, they do not provide Quality of Service (QoS).

**Fig 3:**
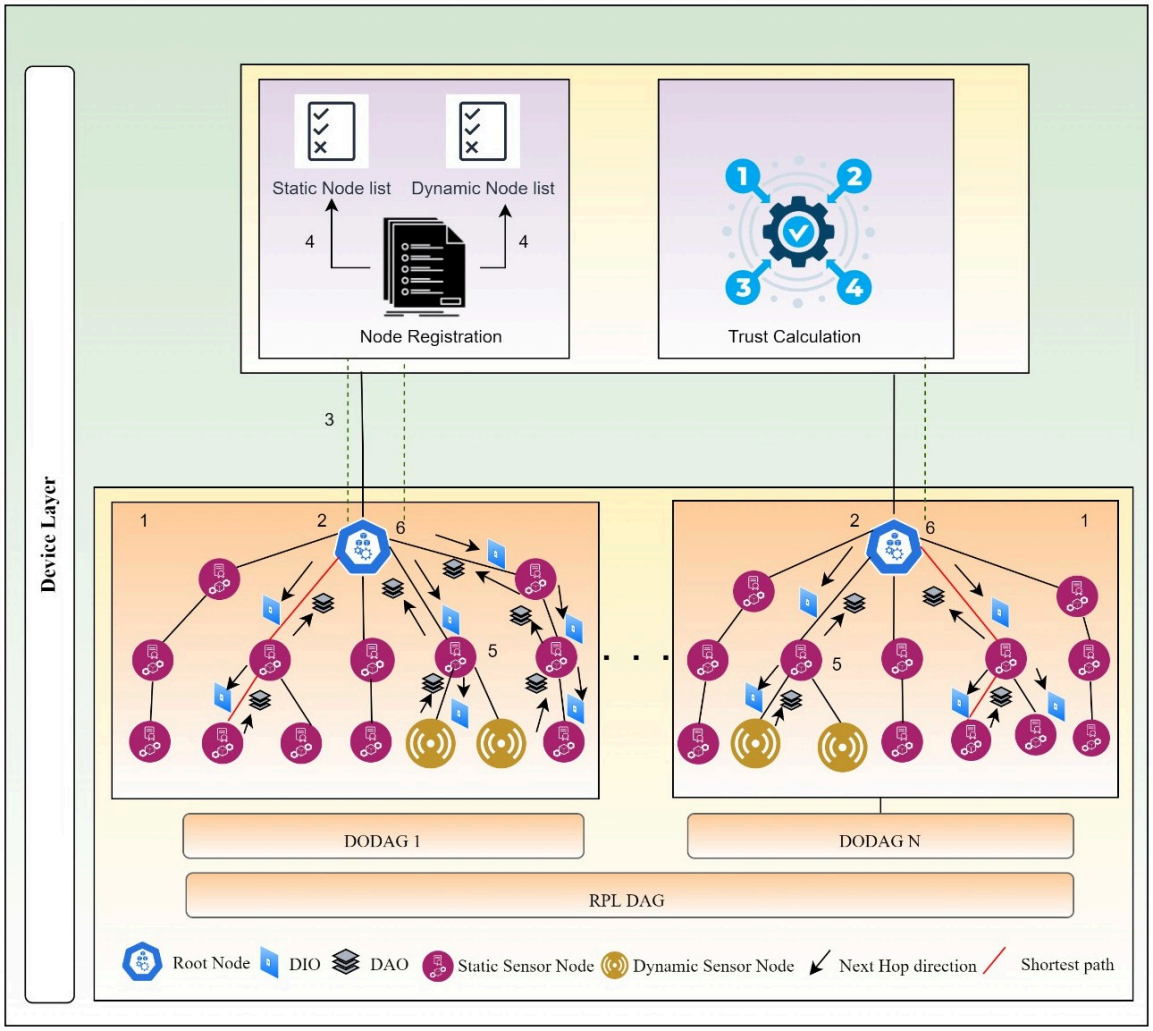
RPL DODAG, Sub-DODAG, and RPL instances.

**Fig 4:**
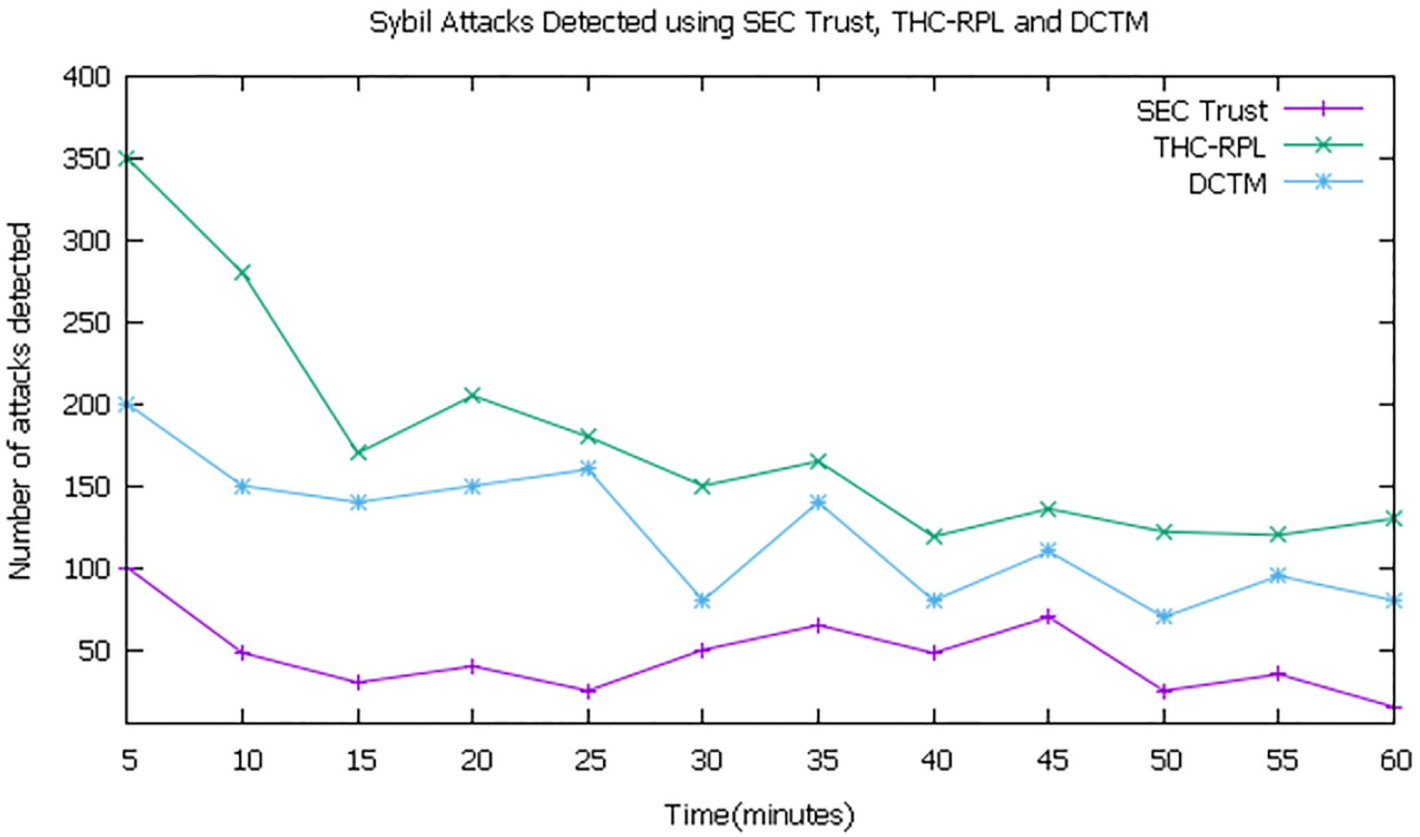
The non-storing and storing modes of RPL.

RPL uses the root node range to determine the location of each node in the DODAG. A complete DODAG is called an instance of RPL. DODAG-ID is IPv6 unique identifier it is used to determine DODAG uniquely in an RPL instance, as shown in Fig 3. A certain change in DODAG results in a change in topology representing the version number of the DODAG [21]. A DODAG topology is formed in a way root node starts sending DIO messages to all nodes. The root node determines its location in all the nodes. Each node at each level of the receiver routers records the path and all the paths for each node involved. These nodes then propagate DIO messages, and in this way, the whole topology is built. The preferred parent node at the development of DODAG will be chosen as the default path to the root node in root formation upwards. While in downward routes, nodes emit and propagate the DAO control message towards the root node using the parent node [20]. The RPL has two modes: a non-storage mode and a storage mode. RPL routes messages to lower levels in a non-storage mode based on IP source routing, as shown in Fig 4.

The traffic goes to the root node and from the root node responsible for sending this traffic to a destination using the routing of the source. While in storing mode, routing towards lower levels is based on destination IPv6 addresses. Every node in DODAG has information about the sub-DODAG and maintains the downward routing table of sub-DODAG. Using this information, traffic is routed towards the destination. In this case, traffic moves upwards, but when it reaches the common ancestor node of source and destination, traffic is transmitted via this node. RPL also provides Peer to Peer (P2P) traffic [22].

### 3.3 Sybil attack

**Fig 5:**
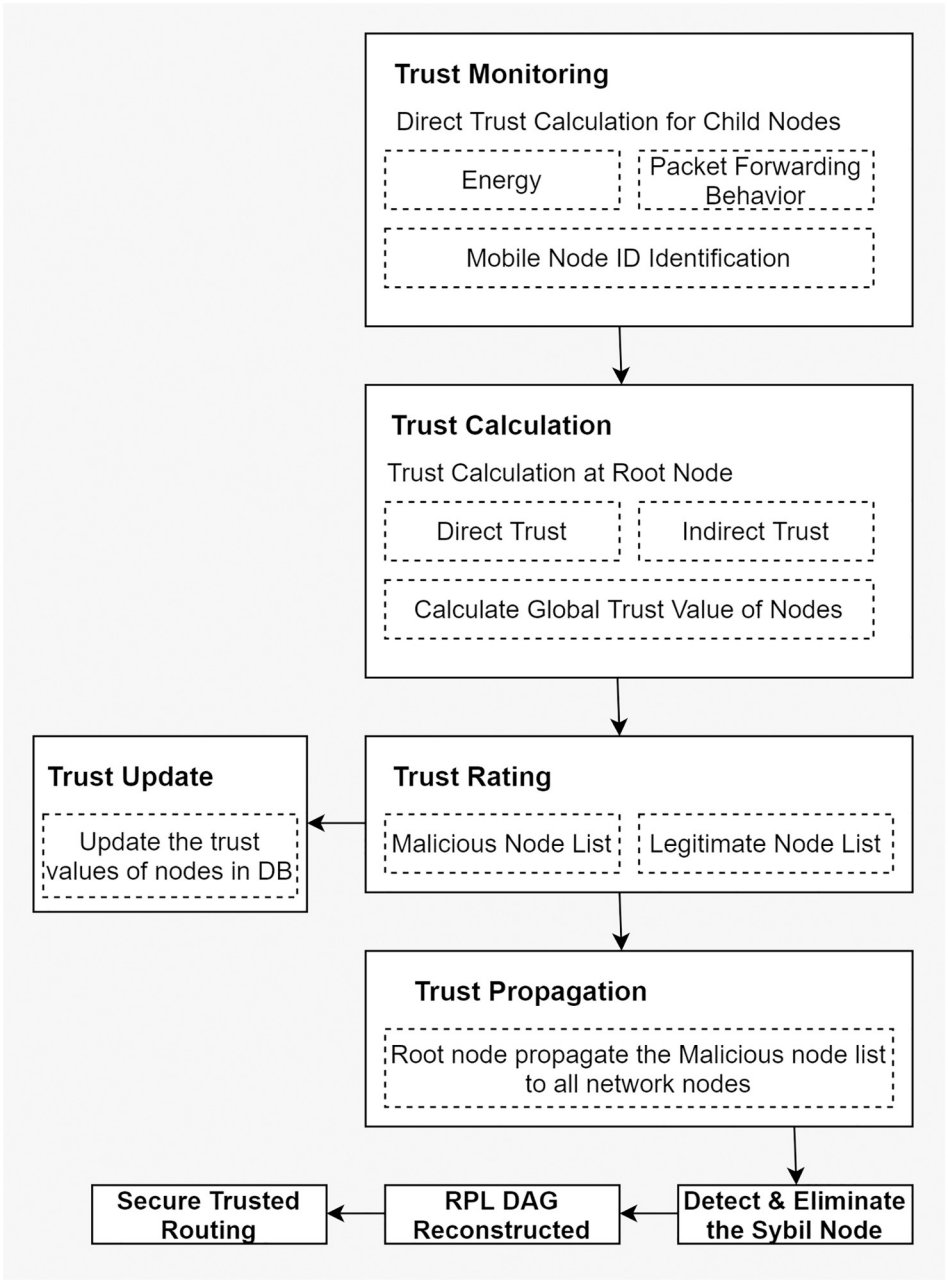
Types of Sybil attack.

A Sybil attack is a form of attack in which malicious nodes take advantage of their neighbor nodes by observing their behavior and stealing their identities or fabricating several logical new identities on the same physical node. The main aim of this type of attack is to influence the entire network without physical nodes being deployed [23]. It is categorized into three groups SA-1, SA-2, and SA-3, according to the relations with its neighbor and mobility component of the Sybil nodes [24]. In SA-1, malicious nodes start connecting with the Sybil community, as shown in Fig 5. In this type, Sybil nodes make tight connections with other Sybil nodes. SA-1 type cannot make tight or strong connections with honest or legitimate nodes. It shows the number of connections between Sybil nodes and legitimate nodes. This attack usually exists in the sensing domain, i.e., mobile sensing systems. The main purpose of this attack, for example, in a mobile sensing context, type SA-1 generally makes fake or forges sensing data and may alter the aggregated data indirectly. Sometimes this action of the SA-1 attack makes it indistinguishable from normal users [25].

In the SA-2 attack, the Sybil node makes connections with other Sybil nodes, as in the case of SA-1, and makes connections with legitimate nodes. Malicious nodes will be spread among honest nodes in this attack. It is very difficult to detect this attack since Sybil nodes have close ties with valid nodes. The main concern of this type of attack in RPL networks is disrupting the topology of routing and compromising any reputation-based system. In SA-3, this type of attack is very critical, similar to the SA-2 attack, but nodes are not fixed in this case, as they may move. Mobility indicates there are weak connections with other neighbor nodes. The concerns of this attack are similar to the SA-2 attack; however, it is very difficult to identify because nodes are mobile [24]. RPL faces a large vulnerable surface internally. Attackers take advantage of the RPL’s internal vulnerability and launch attacks such as Sybil, black hole, selective routing, or grayhole. One of the most vulnerable internal attacks is a Sybil attack. Although other internal attacks also degrade the network performance, the Sybil attack has serious consequences.

**Fig 6:**
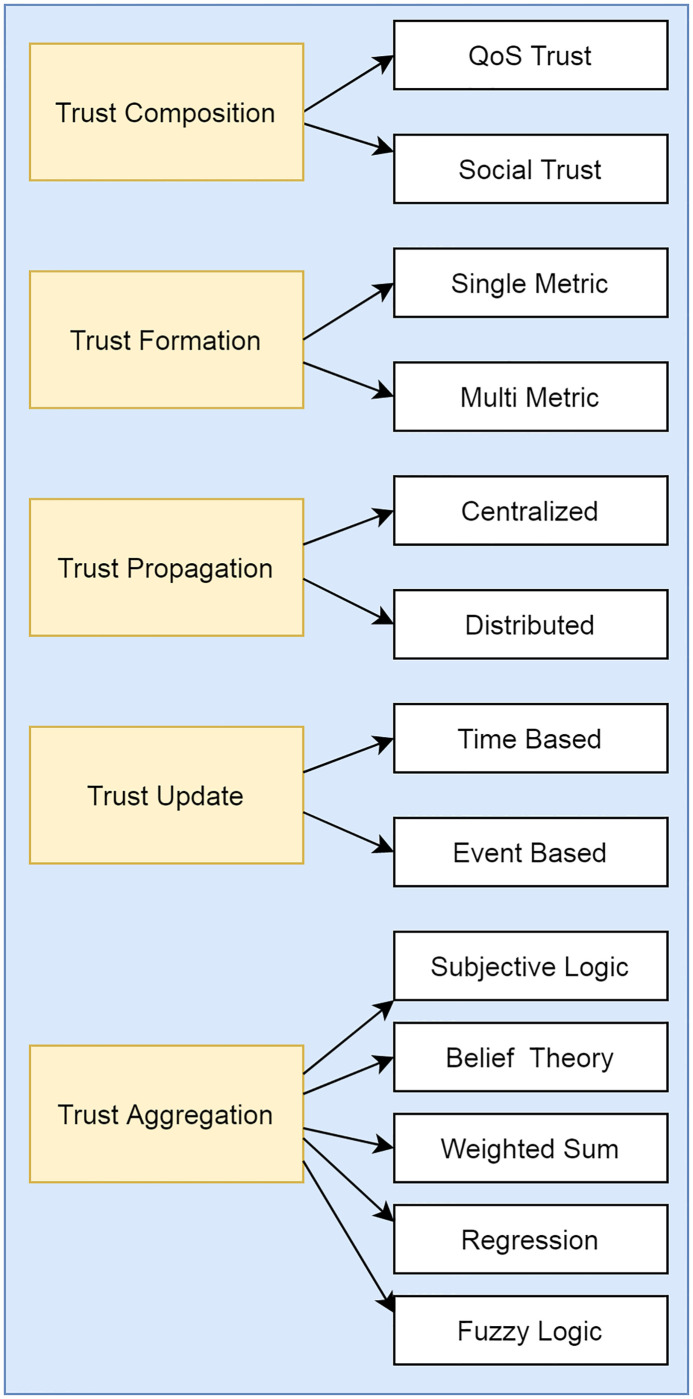
Sybil attack in RPL.

**Fig 7:**
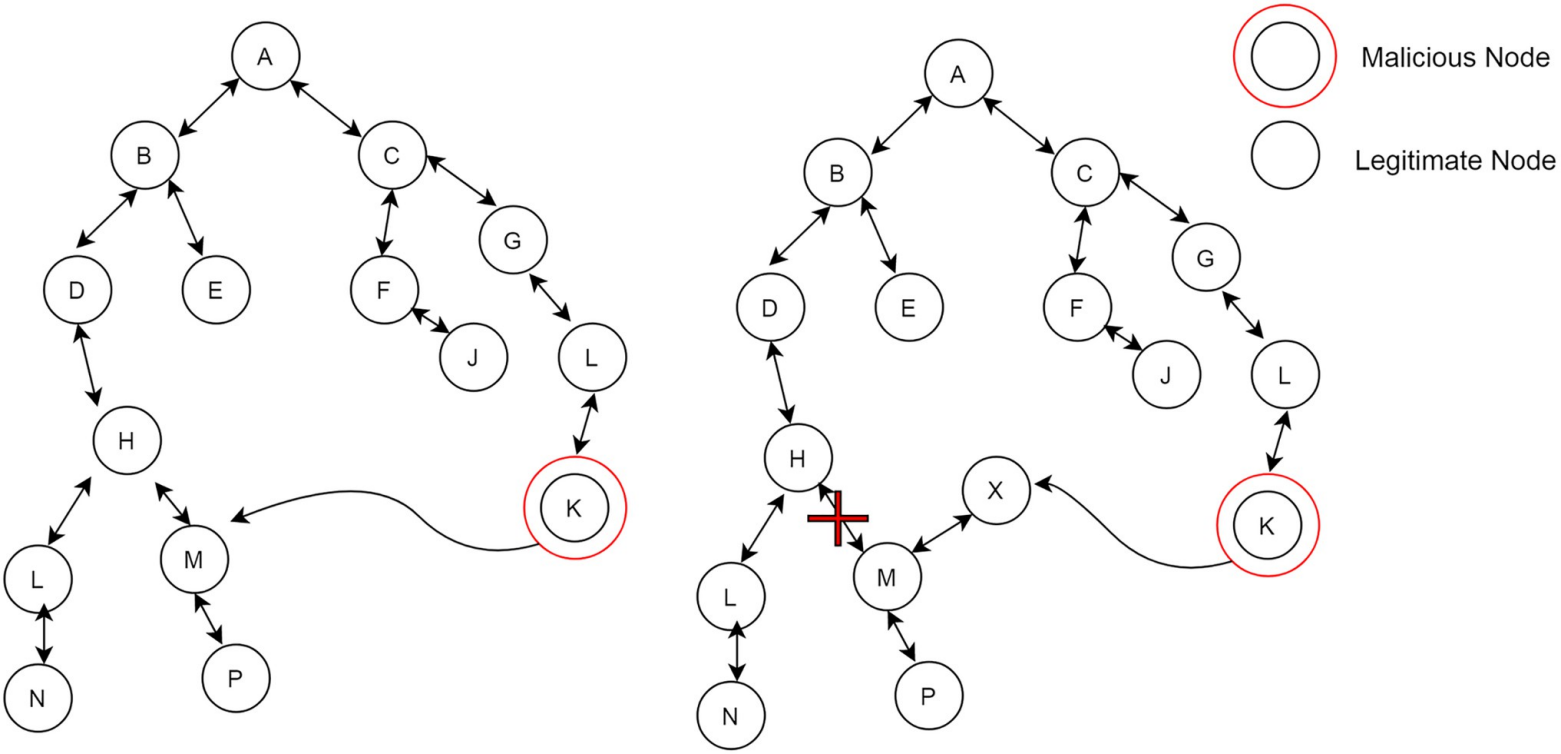
Sybil attack in RPL.

The attack model considered in this research is a mobile Sybil attack. Attacker, while moving in the network, fabricates new identities. In Fig 6 in which a malicious node moves from one point to another point. When it reaches the destination, it fabricates a new identity. A new identity appears as a legitimate node to other nodes of the network. Continuity of this process appearing new identity results in depleting the energy of the low power lossy network devices. While in the second scenario of attack, malicious nodes observed the behavior of their neighbor nodes and stole their identities. The attacker uses several identities on the same physical node to monitor the network without actual nodes being installed. While moving in the network attacker node uses the stolen identity as shown in Fig 7 to disrupt the network topology that, results in the loss of packets of legitimate nodes in the network and also depletes the energy of the nodes. In the Sybil attack, malicious node shows new or stolen identities with lower rank because child nodes try to make them a parent because these Sybil identities have a lower rank than their actual parents. In this way, a malicious node gets the information of these nodes and carries out the malicious activity for which they are in the network. The presence of these Sybil identities degrades network performance because legitimate nodes do not get the required resource.

### 3.4 Threat model and security analysis

**Fig 8:**
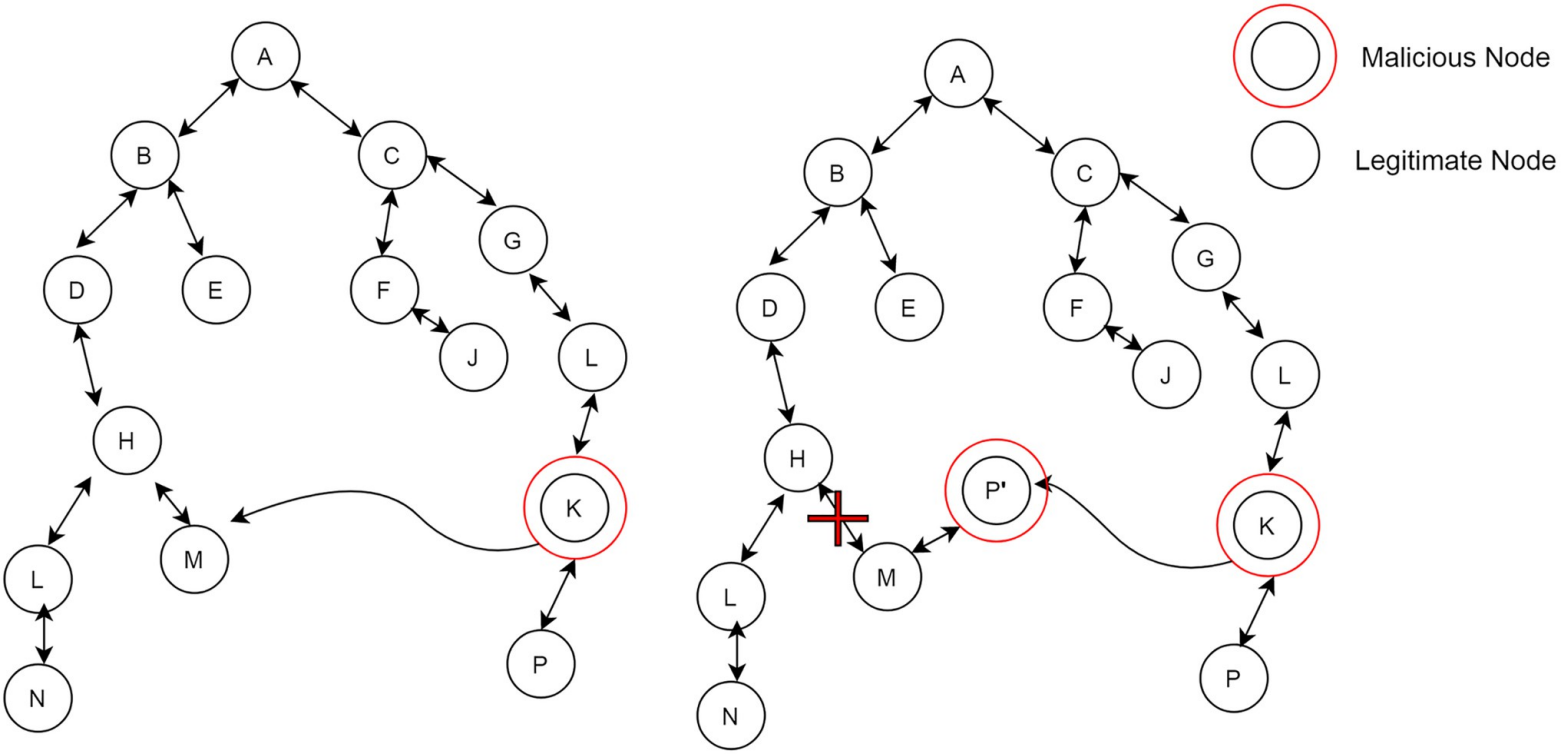
A Sybil attack scenario in smart healthcare domain.

Emerging advancements of technology in medical fields help the patients to overcome different medical conditions. Fig 8 presents a Smart Hospital (SH) system. All doctors, nursing staff, and administrative staff in SH are connected to the system with smart apps. Where all patient details (s) are shared with the corresponding persons (e.g., patient medical details are shared with doctors, while their medication details are shared with staff nurses)—using different sensors and timely taking of procedures results in fast recovery. In the given figure, different devices and sensors are used to collect a patient’s vitals and forward them to the server or system for further correspondence and treatment. These sensors communicate with each other and help the medical staff take immediate action. These sensors working are looking fine; however, due to technological evolution, the data from these sensors go to some remote or local server through the Internet to keep the history of the patient. Therefore, security concerns cannot be ignored here [26]. Initially, the data is collected from patients and surroundings via different sensors. Different sensors and attached to the patient body to measure body temperature, oxygen level, room temperature, humidity, and heartbeat through a wireless link. The data is further sent via other such sensors.

Once data reaches the router, it will forward that data to the server. If a node is compromised, launching a Sybil attack; can send fake identities to other sensors. Under Sybil attack, sensors duplicate the identities or create new identities, making the data for medical treatment ambiguous. Due to ambiguity, the medical staff can face trouble in treating the patient; therefore, in that scenario, this kind of attack might have some serious life-threatening conditions [27]. As shown in Fig 8, All the traffic is towards the malicious node, which can create fatal consequences, such as a DoS attack, packet dropping, and packet delay. To cater to these conditions, we proposed a scheme that successfully detects the attacker nodes and makes the network work legitimately. In this regard, Definitions 0.1–4 represent nodes’ fabrication, compromising nodes, selection of nodes by an attacker, and how to cater to Sybil attacks, respectively.

Let there are *N* number of connected devices in the network. Let *N*_*I*_ be a set on nodes’ identities, where *N*_*I*_ = {*id*_1_, *id*_2_, *id*_3_, …, *id*_*n*_}. To launch a Sybil attack, the attacker have to take possession of a valid identity set *N*_*I*_. Let *N*_*s*_ be a set of Sybil identities, where *N*_*s*_ = {*S*_*y*_1, *S*_*y*_2, *S*_*y*_3, …, *S*_*y*_*n*} and *N*_*s*_ < *N*. *N*_*s*_ can be a result of compromised or fabricated node.

**Definition 0.1**. *The node fabricating process can be defined as a process of possessing a node, such that each S_y_i ∈ N_s_. Where S_y_i ∈ {id_m_n, id_m_x} and ∈ N_I_. They represent the minimum (id_m_n) and maximum (id_m_x) identity range in N_I_, respectively*.

Node fabrication is the most straightforward method of obtaining Sybil identities when the machine-to-machine transmission is unprotected. Sybil nodes may be generated at random by the attacker in this instance. The following measures are often used to avoid the fabrication of nodes in most sensor-equipped networks: a) controlled network limits, ii) surrounding nodes are restricted, and iii) each neighboring node has a distinct frequency channel for communication [28, 29]. Without these limitations, an attacker may only hack or take honest nodes from the network environment, provided the communication between the devices is secure [30].

**Definition 0.2**. *The process of node compromise is possessing a set of Sybil nodes S_t_ for all S_y_i ∈ S_t_, such that S_y_i ∈ {id_m_n, id_m_x} and ∈N_I_*.

**Definition 0.3**. *Let The neighboring node set be A_j_(id_i_) for node id_i_, such that A_j_(id_i_) = id_j_ ∈ N_I_ and DS_T_(id_i_, id_j_ ≤ RD_s_). DS_T_(id_i_, id_j_) is the distance between id_i_ and id_j_, and RD_s_ represents the IoT devices’ communication radius*.

A Sybil node can conflict with the set of nearby nodes of a Sybil identity in a network if an attacker chooses nodes at random. If, on the other hand, an attacker aims to compromise nodes that are part of the network, then it may deliberately select the proximate neighboring nodes of the compromised nodes. A Sybil node may be deployed by the attacker using the compromised ones of nearby nodes without affecting the attacker’s network. Also, compromising legit nodes on the network may bypass all of the network’s security features.

**Definition 0.4**. *Identifying the malicious nodes can be ensured by assuming that R_n_ is the trusted root node and serving as a verifying benchmark. Let DT be the Direct Trust, IDT be the Indirect Direct Trust, and C_T_ be the the total number of nodes in the network, where C_T_ = {C_1_, C_2_, C_3_, …, C_n_}. Let C_E_ be the Energy of Child Nodes and P_T_ be the total number of data packets send, where P_T_ = {P_1_, P_2_, P_3_, …, P_n_}. Let C_DT_ be the Direct Trust of the Child Nodes, and C_IDT_ be the Indirect Direct Trust of the Child Nodes (where the full functionality of the child node is performed using Algorithm 1, explained later in the paper). Let C_DT_ ≥ t, where t indicates the threshold value of 0.6 for trusting C_i_. To compute the value of C_DT_, consider C_T_ and C_E_ while sending the n number of P_T_ to the neighbor node. Initially, all nodes are trusted after some communication. Once the communication has started, we need to identify the malicious nodes for secure data transmission. If C_DT_ satisfies t, let the communication continue. If it does not follow t, perform Algorithm 2 (explained later in the paper). Let R_n_ check the identity C_i_ and C_IDT_ from the child node. If both satisfy t, the communication continues; otherwise, R_n_ declares it as a malicious node and reconstructs the DAG. Therefore, if {C_T_, R_n_}≥t, the C_i_ is an eligible node and meets the condition and is a legitimate node for secure data exchange*.

Table 1 represents a summary of nomenclature used in the paper.

**Table 1:** Summary of nomenclature.

Symbol	Meaning
*N*	Number of Connected Devices
*N*_*I*_	Node Identities
*N*_*s*_	Sybil Identities
*S*_*y*_ *I*	Sybil Identities
*A*_*j*_	Neighbor Nodes
*R*_*n*_	Root Node
*DT*	Direct Trust
*IDT*	Indirect Direct Trust
*C*_*T*_	Total Number of Child Nodes
*C*_*E*_	Energy of Child Nodes
*P*_*T*_	Total Number of Packets
*C*_*D*_ *T*	Child Node Direct Trust
*C*_*I*_ *DT*	Child Indirect Direct trust
*C*_*T*_	Total Number of Child Nodes
*C*_*E*_	Energy of Child Nodes
*C*_*T*_	Total Number of Child Nodes
*P*_*T*_	Data Packets Sent
*C*_*T*_	Total Number of Child Nodes
*t*	Trust Threshold Value
*NCN*	Network Child Node
*BR*	Border Router
*TMNs*	Trust Monitoring Nodes
*NCN-ID*	Network Child Node Unique Identity
*CMN*	Child Mobile Nodes
*DT*	Direct Trust
*IDT*	Indirect Trust
*EXT*	Expected Transmission Count
*SP*	Sent Packets
*FP*	Forwarded Packets

### 3.5 Trust-based security

A relationship between two parties (trustor and the trustee) could be defined as trust. On behalf of the trustor, the trustee carries out his activities. Trustor evaluates the trustee based on how many trustees fulfill the activities of the trustor. Usually, in social science, the concept of trust has been used broadly and imputed as the relationship among objects, persons, and entities. To evaluate the node’s trustworthiness, the trust-based mechanism is a nominal area of research. A trustworthy node is evaluated by the observation of the behavior of this node by a neighbor node. Characteristics such as reliability, confidence level, integrity, belief, and dependability determine the node’s trustworthiness. These properties or characteristics are typically empirically quantified and cumulatively aggregated into trust value based on which the node is evaluated either as ‘trusted’ or not. This trust value would represent the node’s reputation in the network. Trust values determine the positive or negative behavior of the node observed by the neighbor nodes over a while based on direct or indirect interaction of the node with their neighbor node. Trust-based management in IoT has been proven and illustrated as an important idea when building a stable and secure IoT network configuration. A trust-based management system plays a crucial part when the network is expanding that cannot be managed by the central authority. Trust value determines the trustworthiness of the node and QoS (e.g., assistance in selecting the optimal and secure route) a node provides to its neighbor node [24].

**Fig 9:**
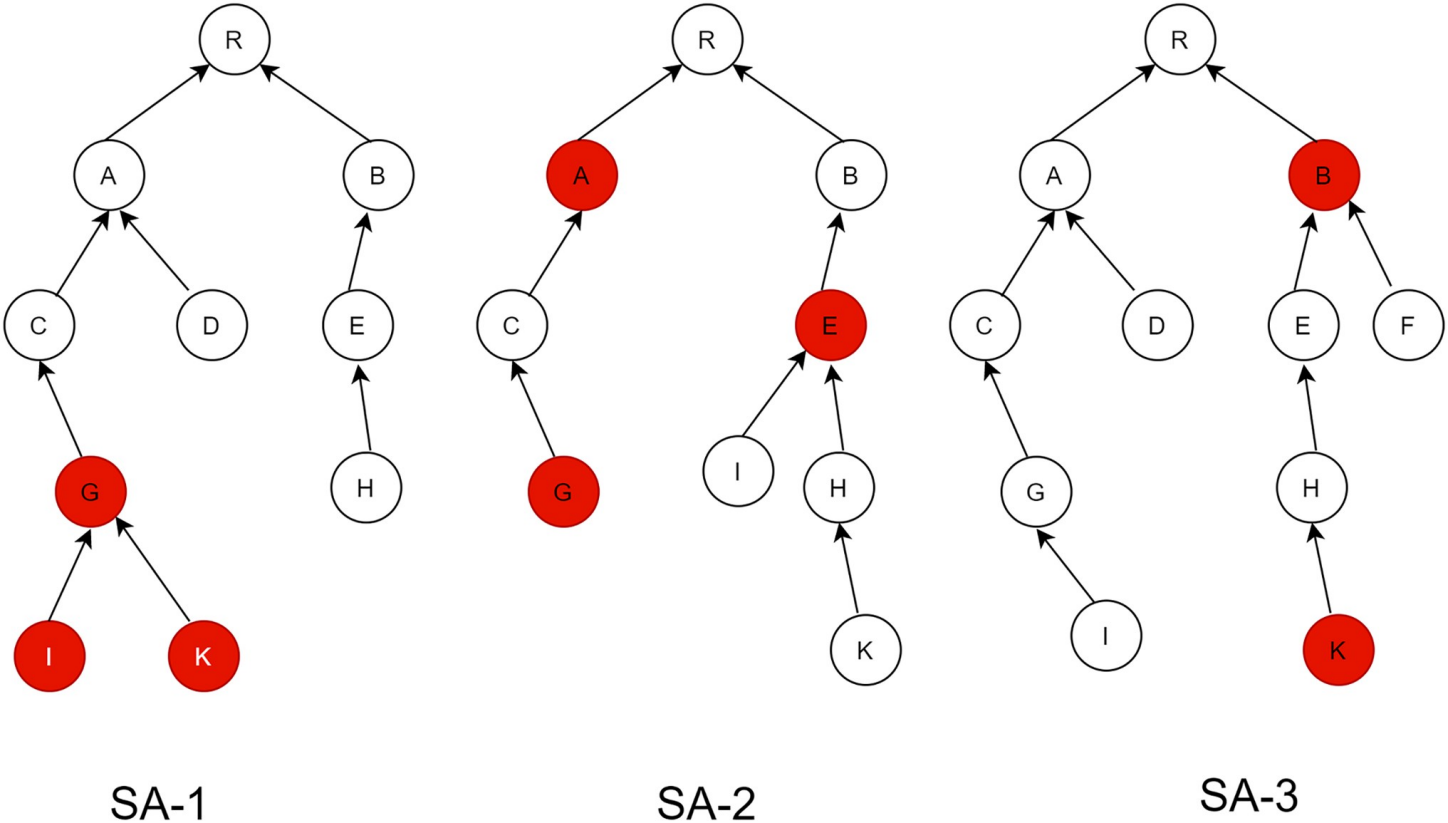
Trust computation architecture.

A Trust computational model consists of five steps, discussed below:

1. Trust composition: refers to the components involved in the computing of trust. QoS and social trust are the building blocks of trust composition. QoS trust determines the degree of belief in the IoT device. It applies to the node’s ability to cooperate, be efficient, be competent, and complete tasks. The evaluation parameters for calculating QoS are energy consumption, end-to-end packet forwarding ratio, and packet delivery [31]. Social trust depends on the social relationship between the possessors of the IoT devices. It is calculated by closeness, connectivity, honesty, and unselfishness. The architecture of the trust-based model is shown in Fig 9.
2. Trust Propagation: refers to how to propagate trust composition through IoT devices. There are two methods to propagate trust; the first one is distributed, and the second is centralized. In the distributed approach, the nodes are considered based on their direct interaction with their neighbors to propagate trust without any centralized body. The centralized scheme requires a centralized body such as a physical cloud.
3. Trust aggregation: refers to proof of the trust obtained from the participating peers by self-observation or feedback. There are many trust aggregating mechanisms such as weighted sum, fuzzy logic, belief theory, analysis of regression, and Bayesian inference.
4. Trust Update: refers to when to update the trust values. Generally, there are two ways to update the trust: Event-driven and the second is time-driven. In an event-driven approach, trust is updated whenever any transaction or event is made. In contrast, time-driven based on the evidence (direct trust or indirect recommendations) are accumulated periodically and using trust aggregation to calculate this for trust updating.
5. Trust Formation: refers to overall trust formation. Usually, trust formation consists of a single trust metric or multiple trust parameters. Single trust metrics should consider only one trust metric, whereas QoS is typically considered the most important property for calculating trust. At the same time, multiple trust parameters consider a range of properties to calculate trust value, such as honesty, energy, and unselfishness.

## 4. Literature review

Airehrour et al. [42] suggested a trust-aware RPL Routing Protocol. They used direct and indirect trust to mitigate Sybil and Rank attack. The proposed scheme involves nodes’ monitoring (periodic or reactive), a trust rating process, and trust backup. However, all the trust calculations are done at the node level, that causes depletion of nodes’ energy. It does not consider the uncertainty of recommendations. Hashemi et al. [8] discuss DCTM-IoT. This new trust-based RPL protocol considers the mobile environment of IoT nodes and solves the security problem under the mobility of IoT nodes. The proposed model is a comprehensive multi-dimension (calculating the trust using three dimensions). The dimensions considered are p2p communication, quality of service, and contextual information. These dimensions have further sub-dimensions, which make this model highly dynamic. This model is not reserved for these measurements; it also considers direct trust and neighboring confidence measurement recommendations. A novel objective function (OF) is proposed by integrating the trust-based model into the OF of RPL. However, the sink mobility is not considered; all the processing of trust calculation is done at the node level with a huge number of parent changes for best-path calculation. Djedjig et al. [32] determine a large vulnerable space of IoT to perform different attacks. SPLIT, a secure and scalable routing protocol for IoT networks, is proposed in this paper. This approach uses the attestation method concept. The attestation method involves ensuring the integrity of the software. The proposed approach is integrated into the DAO control message of the RPL and compared with the standard RPL protocol. However, the extra computation layer introduction in the RPL DAO messages is not energy-efficient.

Medjek et al. [33] proposed a Metric-based RPL Trustworthiness (MRTS) protocol for the RPL. The author introduces a new metric, ERNT, to select a node worthy of trust while building the route from source to root node. This metric calculates the trust at each node of the network, including selfishness and energy. They addressed the Self Promotion, Ballot Stuffing, and Bad mouthing attacks. However, all the processing is done at the node level, that causes DIO messages overhead. Hashmi et al. [34] addressed Rank, Sybil, and Blackhole attacks. They introduced a Multi-Fuzzy Dynamic and Hierarchical Trust Model (FDTM-IoT). It considered different trust matrices such as QPC, QoS, and Contextual information. Every metric is calculated using its sub-matrices then a single difuzzified value is obtained, which determines the level of trust. However, it is not energy efficient delay. Conti et al. [35] evaluated the performance of the RPL protocol Mobile Sybil (SybM) attack. However, the proposed RPL is for static topology. This attack affects identity, and mobility and floods the network with fake messages from different locations. In addition, the whole trust-based intrusion detection system is in DIO, which poses extra overhead on the node, ultimately resulting in the energy depletion of nodes. Furthermore, this intrusion detection system is not evaluated through simulation. Another trust model is presented in [36] for distributed computing. It uses direct trust, which is evaluated over the nodes based on the number of communications. If the direct trust value satisfies the threshold, the node is considered legitimate; otherwise, it is malicious. However, the proposed model is not energy-efficient in resource-constrained IoT networks. In addition, the mobility of nodes is also not addressed.

Medjek et al. [6] discussed the internal security threat on RPL performance. Introducing the attack model in which Sybil nodes are mobile, they performed malicious activities by creating different Sybil identities by changing the position using their mobility aspect. The paper analyzed the performance degradation in the presence of the mobile Sybil nodes. With the presence of mobile Sybil node rate of packet delivery drops, and control overhead message increases, resulting in the energy depletion of the energy constraint nodes. Farooq et al. [37] author uses different metrics to illustrate multi sink routing protocols in the wireless sensor network. These protocols are based on Low power lossy network RPL. Using various metrics such as available bandwidth, MAC layer queue occupancy, latency, and expected transmission count (ETX) together with the shortest hop count metric. The objective functions of the RPL use different metrics based on a greedy approach or an end-to-end basis. The proposed protocol using different metrics increases the packet delivery ratio by up to 25. It reduces the number of re-transmissions by up to 65 compared to the standard version of the RPL using only hop count metrics for routing decisions. Three kinds of objective functions are used in the proposed approach, which increases the algorithm’s complexity. Table 2 provides a summary of comparisons made among different state-of-the-art techniques proposed to mitigate internal attacks.

**Table 2:** State-of-the-art comparison.

Reference	Technique	Attack addressed	Weakness	Evaluation Parameters
[7]	Trust	Rank, Sybil	Not energy efficient, Single point of failure, Uncertainty of recommendations	Packet Loss Ratio, Attacks Detected
[8]	Trust	Rank, Sybil, Blackhole	Large number of parent change	Average parent change, Packet loss ratio, End-to-End delay, Average energy consumption
[33]	Trust-Based IDS	Sybil	Trust Platform module, Extra computation layer	Control overhead, Energy Cost, Packet Delivery Ratio
[35]	Attestation Method	Blackhole, Sybil, Wormhole	Extra computation in DAO control message, Not energy efficient	Average packet loss ratio
[32]	Trust	Self-Promotion, Ballot-Stuffing, Bad-Mouthing	Computation and communication overhead	Expected transmission count
[34]	Trust	Rank, Sybil, Blackhole	Not energy efficient, delay, Computation overhead	Packet Loss Ratio, end-to-end delay, Average energy consumption

## 5. Methodology

**Fig 10:**
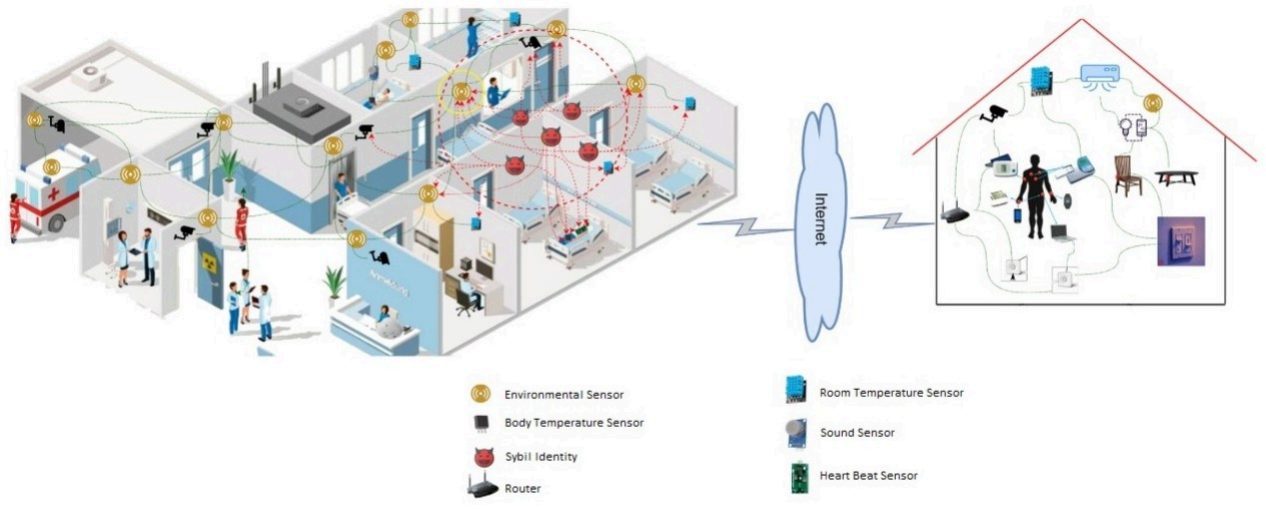
Block diagram of proposed methodology.

**Fig 11:**
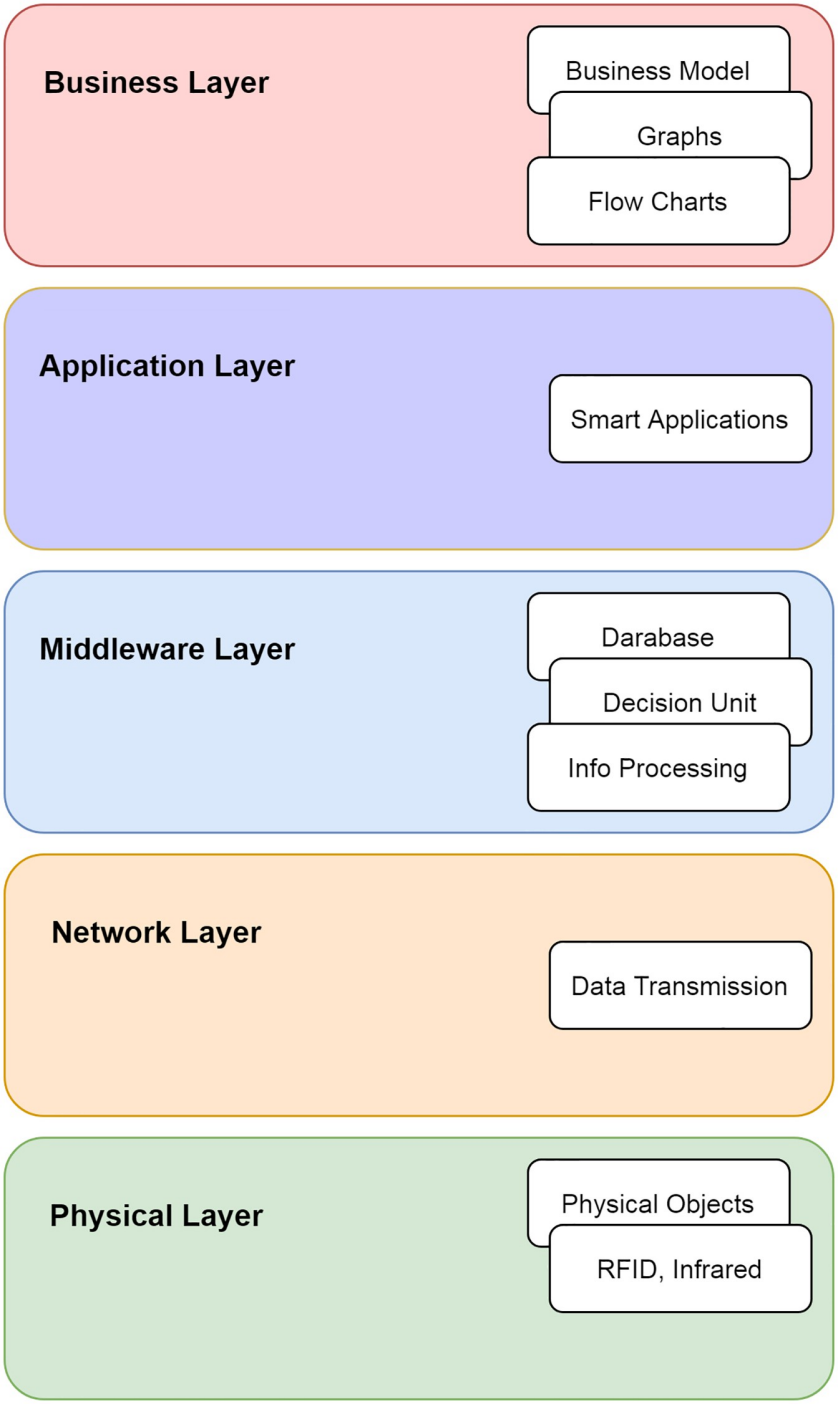
The proposed THC-RPL architecture.

This section gives an insight into the proposed methodology of THC-RPL. Firstly, it discusses the assumptions made during experimentation. Secondly, the proposed system architecture is detailed. Thirdly, it describes the subjective logic trust model, and finally, it articulates the THC-RPL solution, how it works, and the major steps involved. A block diagram of the proposed methodology is illustrated in Fig 10, which is further supported by Fig 11.

The workflow of the proposed THC-RPL is shown in Fig 11. The steps are detailed below:

1. In the first step, the RPL topology is formed. All child nodes send DIO messages, and in response, the root node sends the DAO messages.
2. In the second step, each child node gets registered and assigned a unique ID from the root node.
3. In the third step, the root node creates a list of mobile/dynamic and static nodes.
4. In the fourth step, the root node sends the dynamic node list to all the static nodes in the network to identify the mobile nodes in the network.
5. In the fifth step, child nodes calculate the direct trust of the neighbor node and send it to the root node, where global trust is calculated.
6. In the sixth step, the root node calculates the indirect trust and evaluates the status of the node, either as a legitimate or malicious node. After deciding the nodes’ trustworthiness, it is decided whether a node is genuine or Sybil; and, depending upon the decision, would it be a part of the network or not.

### 5.1 Assumptions

The following assumptions, in connection with the proposed solution design, are made:

- Initially, all nodes of the network are secure, and there is no malicious node.
- The root node or Border Router (BR) is a resourceful device.
- All devices are registered with a unique identifier with the root node.
- Devices other than root may or may not be mobile; the root will remain static.

### 5.2 System architecture

**Fig 12:**
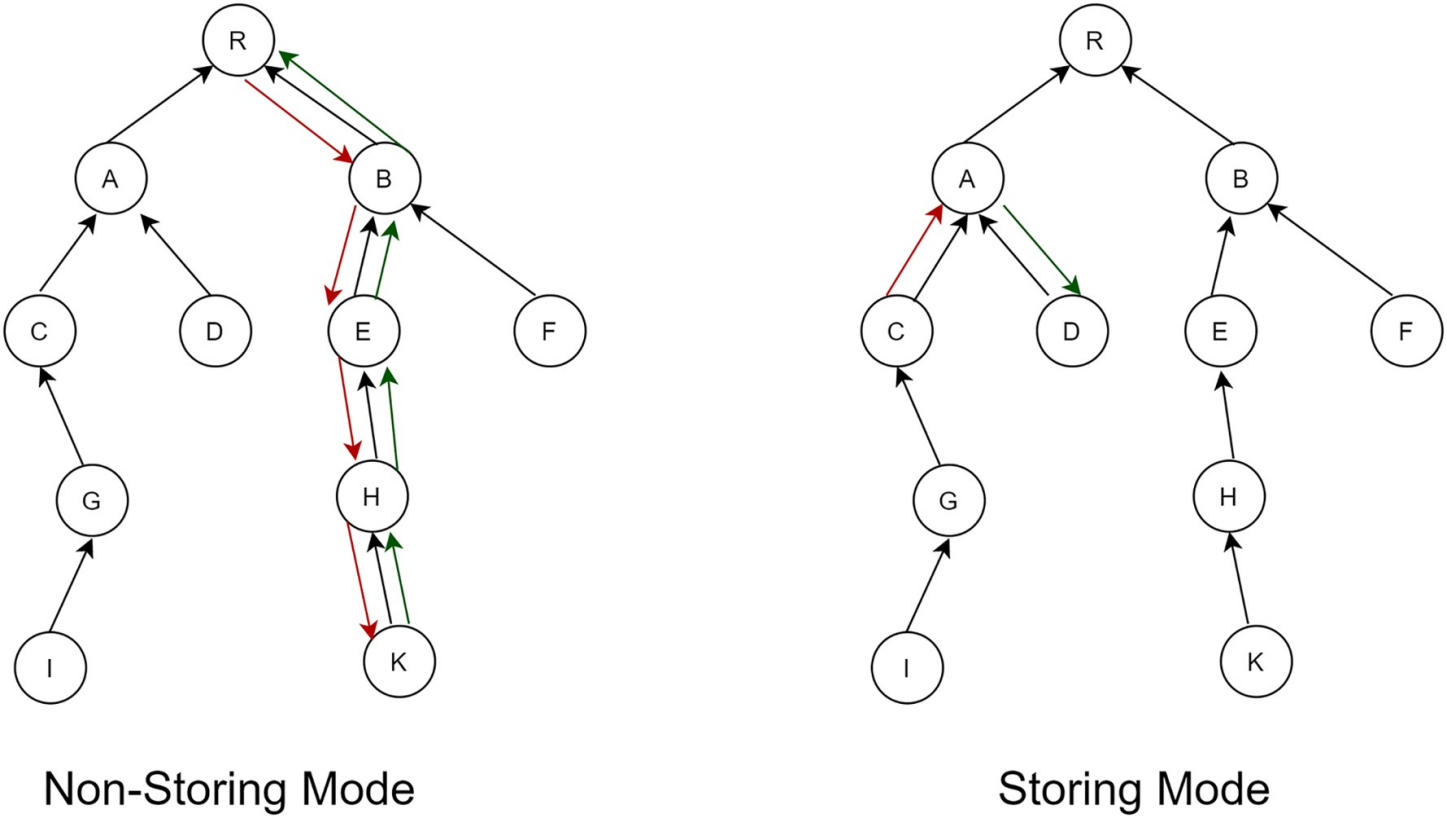
Sybil attack in RPL.

The architecture of the proposed methodology consists of a root node, and the rest of the nodes are child nodes. The root node is a resourceful device with more computation power than other devices, while others have less computation power. The devices communicate with each other using the THC-RPL protocol, as shown in Fig 12. The proposed method uses the subjective logic model for trust computation, described next.

1. Subjective Logic Trust Model
  This model computes trust based on the behavior of nodes. This model was firstly suggested by Josang [41]. It determines the world’s subjective beliefs and is represented as ‘opinion’. An Opinion can be calculated as a secondary uncertain probability measure. In IoT, nodes may be mobile and stationary. In the case of stationary nodes, giving an opinion on the nodes’ trust, the trust becomes solid because nodes have more stable connections. While in the case of mobile IoT devices, giving an opinion about a node’s trustworthiness may be uncertain because it does not have stable and long connections with neighboring nodes. In mobile cases, less evidence about a node’s trustworthiness is available due to the nature of the mobility challenge [5, 6, 38]. Most of the traditional probability models used in trust computing do not consider the uncertainty factor when giving an opinion about a node’s trustworthiness. To meet such needs, belief, disbelief, and uncertainty are provided by subjective logic. Subjective logic maps evidence space and domain of perception (opinion space) by turning trust.
  On RPL, the impacts of the internal attacks can be devastating. If the network devices are mobile, it becomes more difficult to detect and mitigate internal attacks. Consider a Sybil attack where an inside network node becomes malicious and creates new identities. New or stolen Sybil identities appear as normal nodes. These Sybil identities send control messages for joining the RPL DAG. It results in losing the power for the already existing nodes. Therefore, mitigating the consequences of a Sybil attack is crucial in RPL. Moreover, designing new techniques is inevitable to detect and mitigate these internal attacks. However, existing techniques have several weaknesses. They are computationally expensive, energy costly, limited to sensor networks or Adhoc networks, or essentially designed for non-mobile nodes. Different schemes have been proposed while considering the IoT devices, and different schemes have been proposed [23]. What makes Sybil attack more difficult to detect in a mobile case is that malicious nodes fabricate new identities and steal the identities of the neighbor nodes. These new identities appear as legitimate or malicious nodes, observe their neighbors’ behavior and create a stolen identity of already existing nodes making detection challenging. Hence, to detect and mitigate the Sybil attack in a static and mobile scenario in RPL-based networks, this paper proposes a Trust-based Hybrid Cooperative RPL (THC-RPL) protocol.
  The THC-RPL protocol considers the trustworthiness of neighboring nodes and identity modules. In the case of static nodes, trustworthiness includes two metrics: energy consumption and packet forwarding behavior. While in mobile cases, it also considers the node’s trustworthiness and checks the identity of a mobile node. Every node monitors and calculates the trust level based on two metrics of the one-hop neighboring node. Using the trust level of nodes with their neighboring nodes, the root node calculates the global trust of the node to check its credibility. The typical RPL protocol uses two types of objective functions OF-0 and MRHOF [8]. However, this study considers the MRHOF and proposes a new objective function. It is so because we use more than one metric to detect and mitigate the Sybil attack. THC-RPL uses the proposed objective function to calculate the trustworthiness of the nodes. Initially, RPL uses its default metric, which is Expected Transmission Count (ETX). This metric considers how many transmissions are required to send a packet. When the DAG of RPL is completely formed, every node observes its neighbor’s behavior for reliable communication.
2. THC-RPL Solution Actors
  THC-RPL consists of Border Routers (BRs), also called root nodes, and the Trust Monitoring Nodes (TMNs). All in-network nodes and Border routers communicate through THC-RPL.
  - Root Node or Border Router (BR)
    It maintains the list of all the Network Child Nodes (NCNs) and the state of the nodes, either a static or mobile node using an NCNs list for authorized access to the network. BR assigned a unique NCN-ID to every child node joining the network. In this way, every node has a unique identifier in the list of NCN. BR uses a flag to maintain the status of each node, whether it is static or mobile. Any node joining the network must first register with the BR and then enter the NCNs list of the root node. It also maintains the list of malicious nodes after evaluating the trust level of NCNs. Root nodes maintain the Child Mobile Nodes (CMNs) list and propagate it to all NCNs to identify mobile nodes.
  - Trust Monitoring Nodes (TMNs)
    every NCN monitors the behavior of their one-hop neighboring nodes. It calculates the trust of its neighbor nodes and informs the BR or Root node through another parent node when the trust value does not meet the threshold value.
3. THC-RPL Solution
  In the proposed work, all the TMNs select their parent using the default metrics ETX. The Rank is calculated on the standard RPL inherent in THC-RPL protocol. The BR node with a rank equal to 1 is chosen as the root node of the network. All other nodes (i.e., NCNs) Rank is higher than the Root node, and they form an inverted Directed Acyclic Graph (DAG). After the DAG is formed, NCNs start communicating. The certain trustworthiness of the node is evaluated based on Direct Trust (DT) and Indirect Trust (IDT) among NCNs and BR, as discussed below:
  - Direct Trust (DT)
    DT determines how trustworthy a node is and how much it fulfills its assigned job. In THC-RPL, DT is calculated based on two metrics: node energy consumption and forwarding behavior. DT is calculated using Eq 1 [39].
    $$\begin{array}{c} DT\left(NCN_{\text{i}},NCN_{\text{j}}\right)=\frac{FPB+\epsilon_{\text{n}}}{2} \end{array}$$ (1)
    ‘FPB’ represents the forwarding packet behavior, and ‘*ϵ*_n_’ shows the change in energy while forwarding the messages. A combined average value gives a DT value of the positivity or negativity of the node.
    - Change in Energy Consumption (AE):
      It determines how much energy is consumed by node B while forwarding messages to node C on behalf of node A. Eq 2 is used for calculating the change in energy consumption.
      $$\begin{array}{c} \epsilon_{\text{n,t}}=p\times \epsilon_{\text{n,p}}\;Where\;\epsilon_{\text{n}=\epsilon_{\text{n,t-1}}-\epsilon_{\text{n,t}}} \end{array}$$ (2)
      Eq 2 shows how much energy is consumed while sending ‘p’ messages. Finally, the difference in energy consumed in forwarding messages in the past and current events shows the energy depletion of the neighboring node.
    - Forwarding Packet Behavior (FPB)
      It determines the ratio of forwarding packets to the sent packets. FPB refers to how many packets B sends to C on behalf of A. Similarly, SP refers to how many packets are sent by node A to B. FPB is calculated using Eq 3.
      $$\begin{array}{c} FPB\left(NCN_{\text{i}},NCN_{\text{j}}\right)=\frac{FP_{\text{ji}}}{SP_{\text{ij}}} \end{array}$$ (3)
      After monitoring the behavior of neighboring nodes based on these two metrics, the DT of the neighboring node is calculated. If the value of the DT meets the threshold, then the trust model increments the positivity of the node. If DT does not meet the threshold, the trust model increments the negativity shown in Algorithm 1. Following the same pattern, all nodes calculate the DT of the neighbor node and transmit it to the root node, as shown in Fig 12. The transmission depends on the value of DT; if the value increments the negativity of the model, then the child node changes its parent and informs the root node actively. The root node checks the NCN-ID and DT then calculates the Indirect Trust (IDT). If at the root node, while calculating the global trust value of the respective node, when the IDT value does not meet the criteria, the ID of the respective node is scanned through the network to check the stolen identity. If a duplicate identity is found, this node falls into the malicious node list. In the case of mobile nodes, the same metrics for DT are calculated by NCNs on receiving a control message from the mobile node, which initially was at x location. After moving to the y location, NCN static nodes check the CMNs list provided by the root node. If this mobile node exists in CMN, changes in energy consumption and forwarding packet behavior are monitored. It informs the Root node if it does not exist and the trust value does not meet the threshold value. If a node steals the identity of any node, it is detected by trust calculating metrics. Moreover, the node is scanned throughout the network if the same identity exists while scanning. This respective node falls in the malicious node list and is removed from the network.
  - Indirect Direct Trust (IDT)
    IDT determines how much a node is trustful by considering the opinions of other nodes, which are DT values of the same node with different neighbors. The root node calculates the global trust periodically and reactively. Periodic transmission of DT value is considered when the network is running smoothly, and after some time, the DT of every node is accumulated from all its neighbors. IDT is calculated as the average value of the trust is calculated. The reactive case runs when the trust model faces negative behavior in which the value of DT of a node increments the negativity of the model. It results in immediate action by informing the Root node. In the case of a new Sybil identity, it checks the identity and calculates the IDT by using the DT of the last event only, which occurs between that malicious node and its neighbors. Algorithm 2 is utilized to find if the under consideration node is malicious. If a duplicate identity is found, the CMNs list is updated and propagated to all NCNs.
    The metrics: forward packet behavior and energy depletion are used to compute the positive and negative interactions among nodes and give an opinion about the trustworthiness of the neighbor node. Based on the values of positivity *p* (i.e., calculated using Eq 4), negativity *n* (i.e., calculated using Eq 5), and uncertainty *u* (i.e., calculated using Eq 6), subjective logic calculates the belief, disbelief, and uncertainty of the node. Every node of NCNs monitors neighbor nodes’ positive and negative events based on these values. The belief, disbelief, and uncertainty is calculated at the Root node. The Eqs 4 to 6 are (used for calculating belief, disbelief, and uncertainty) are taken from [24].
    $$\begin{array}{c} b_{\text{ij}}=\frac{p}{p+n+k} \end{array}$$ (4)
    $$\begin{array}{c} d_{\text{ij}}=\frac{n}{p+n+k} \end{array}$$ (5)
    $$\begin{array}{c} u_{\text{ij}}=\frac{k}{p+n+k} \end{array}$$ (6)
    Where “k” is used as a constant to simplify the computations, its value is set to 0.2 to avoid division by zero [40].
4. Trust calculation
  Subjective logic represents trust as a discrete value between 0 and 1. This value is used as an opinion to describe the trustworthiness of the node, using the parameters of subjective logic, which are belief (*b*), disbelief (*d*), and uncertainty (*u*). Based on these metrics, a weight is calculated about the node’s trustworthiness. The weight calculated should be equal to 1 as shown in Eq 7 [41].
  $$\begin{array}{c} b+d+u=1 \end{array}$$ (7)
  As ‘belief’ shows the probability of how many nodes A can be trusted by node B. Similarly, node ‘disbelief’ represents how much node A is untrusted, and the uncertainty parameter ‘uncertainty’ is not sure that node comes under belief or disbelief. This gap is completed using the void in the absence of b and d.
5. Trust Aggregation
  Trust aggregation is done on the root node periodically and reactively. Suppose NCNs energy consumption metric and forwarding packets behavior of node do not meet the threshold. Then, NCN will inform about the misbehaving node and choose another parent node to send its trust value. The root node calculates the IDT and makes an aggregate based on p and n obtained from the NCNs. In subjective logic, a consensus operator (⊕) is used for trust aggregation.
  The trust value vector of node (*I*) is (*V*_*ij*_(*b*, *d*, *u*)) with respect to node (*J*) and the trust value vector of node(*I*) is (*V*_*ik*_(*b*, *d*, *u*)) with respect to node (*K*) is aggregated as *V*_*i*_ = *V*_*ij*_ + *V*_*ik*_ and computed as shown in Eq 8.
  $$\begin{array}{c} \left(\frac{b_{\text{ij}}u_{\text{jk}}+b_{\text{ik}}u_{\text{ij}}}{k},\frac{d_{\text{ij}}u_{\text{jk}}+d_{\text{jk}}u_{\text{ij}}}{k},\frac{u_{\text{ij}}u_{\text{jk}}}{k}\right) \end{array}$$ (8)
  Here
  $$\begin{array}{c} k=u_{\text{ij}}+u_{\text{jk}}+u_{\text{ij}}u_{\text{jk}} \end{array}$$ (9)
  The aggregated value represents the global trust level of a node.
6. Trust Rating
  Node trustworthiness is evaluated based on the belief, disbelief, and uncertainty value. The value of belief, disbelief, and uncertainty is equal to 1. Subjective logic creates a node rating threshold, as shown in Table 3. The objective of these thresholds is to eliminate the malicious nodes from the legitimate nodes. By using this threshold, only trusted nodes take part in routing.
7. Trust Propagation
  After rating the NCDs, the Root node makes a list of malicious nodes and sends it to the NCNs to eliminate them. It also creates a list of trusted nodes and updates in a database that assures only trusted nodes take part in routing.
8. Trust Update
  There are two methods to update the trust value: periodic and reactive. Both are used in the proposed technique.
  - Periodic:
    THC-RPL uses this technique when the network runs smoothly; positive interactions remain between the NCDs.
  - Reactive:
    When informing the root node about the malicious behavior in this scenario, the reactive response is taken by NCDs.

**Table 3:** Nodes’ rating based on trust values.

Trust Value	Trust Status
0.7–1	Good
0.5–0.6	Fair
0.2–0.4	Poor
0.0–0.1	Not Verified

**Algorithm 1 Calculating the Direct Trust of Neighbor Nodes**.

0 **input**: *CMN*

**begin**

**if** (CMN.ID ∈ NCN.ID) **then**

 // Calculate the Direct Trust (DT) Value Of Directly connected Neighbor Node

  DT = (FPB + *ϵ*_n_) /2

  **if** (DT >= Threshold) **then**

   1.Increment the Positivity Value

   2.Send the DT value periodically to Root Node if NCN.ID matches

  **end**

 **else**

  1. Increments the Negativity Value

  2. Send the DT value Reactively to Root Node if NCN.ID does not match

 **end**

**end**

**Algorithm 2 Sybil attack detection at root node**.

**input**: *DT*

//Direct Trust of all neighbor nodes from Algorithm 1

**begin**

 //Check validity of node using the database containing the list of all nodes registered with Root node

 **if** (NCN.ID ∈ NCN.ID) **then**

  //Calculate Indirect Trust (IDT)

  IDT = (DT_1_ + *DT*_2_ + … + *DT*_*n*_ − 1)/*n*

  // Rate the nodes based on IDT

  **if** (IDT >= Threshold) **then**

   1. Perform the normal operations

  **end**

  // Check the duplicate Identity in the network via Root node

  **if** (IDT < Threshold && NCN.ID IN (NCN.List − NCN.ID) **then**

   1.Declare the respective node as Sybil node

   2.Add it to malicious node list

  **end**

 **else**

  1. Declare the node as Sybil node

  2. Add it to the malicious node list

 **end**

 **return** malicious node list to NCN

**end**

## 6. Evaluation and results

Simulations evaluate the performance of THC-RPL. The simulator used for this purpose is Cooja, which is integrated into the Contiki operating system. The evaluation is carried out using the environment and parameters set in [7, 8]. Using the single metric in standard RPL does not provide internal security in the network. The work in [8, 20] has integrated a security mechanism into the standard RPL using different matrices. Sybil attack is one of the internal security attacks, and existing techniques mitigate this attack using different matrices in the trust computation model. These techniques compute the trust value at the node level. The consequences of computation at the node level include high computation that depletes the energy of low power and lossy IoT network devices. However, the THC-RPL objectives are to reduce the computations at the node level and offload trust computation at the Root node, which is a resourceful IoT device. Ultimately, this objective is to preserve the energy of low-power lossy network devices to work for a longer time. Secondly, it does not use any external device for these trust-related computations. The experiments are performed on 30 nodes, three malicious and mobile nodes in a 3:1 ratio, with the simulation running for 60 minutes. THC-RPL is evaluated based on the “Number of Sybil attack detected,” “Packet loss ratio,” “Average energy consumption of nodes,” and “Average energy consumption of the network”. To evaluate the performance of the work, we considered a smart home environment simulation, where one border router is static, and all the remaining nodes may or may not be mobile. The details of simulation parameters are shown in Table 4.

**Table 4:** Simulation parameters.

Simulation Parameters	Value
Simulation tool	Contiki /Cooja 3.0
Deployment Type	Random position (based on smart home)
Emulated nodes	T-mote Sky
Simulation coverage area	100 m * 100 m
Total number of nodes	30
Malicious nodes	1:10
RX ratio	30-100%
TX ratio	100%
TX range	50 m
Interference range	50 m
Routing protocols	THC-RPL
Simulation time	60 min
Link failure model	UDGM
Mobility speed	0–6.23 km/h

### 6.1 Number of attacks detected

**Fig 13:**
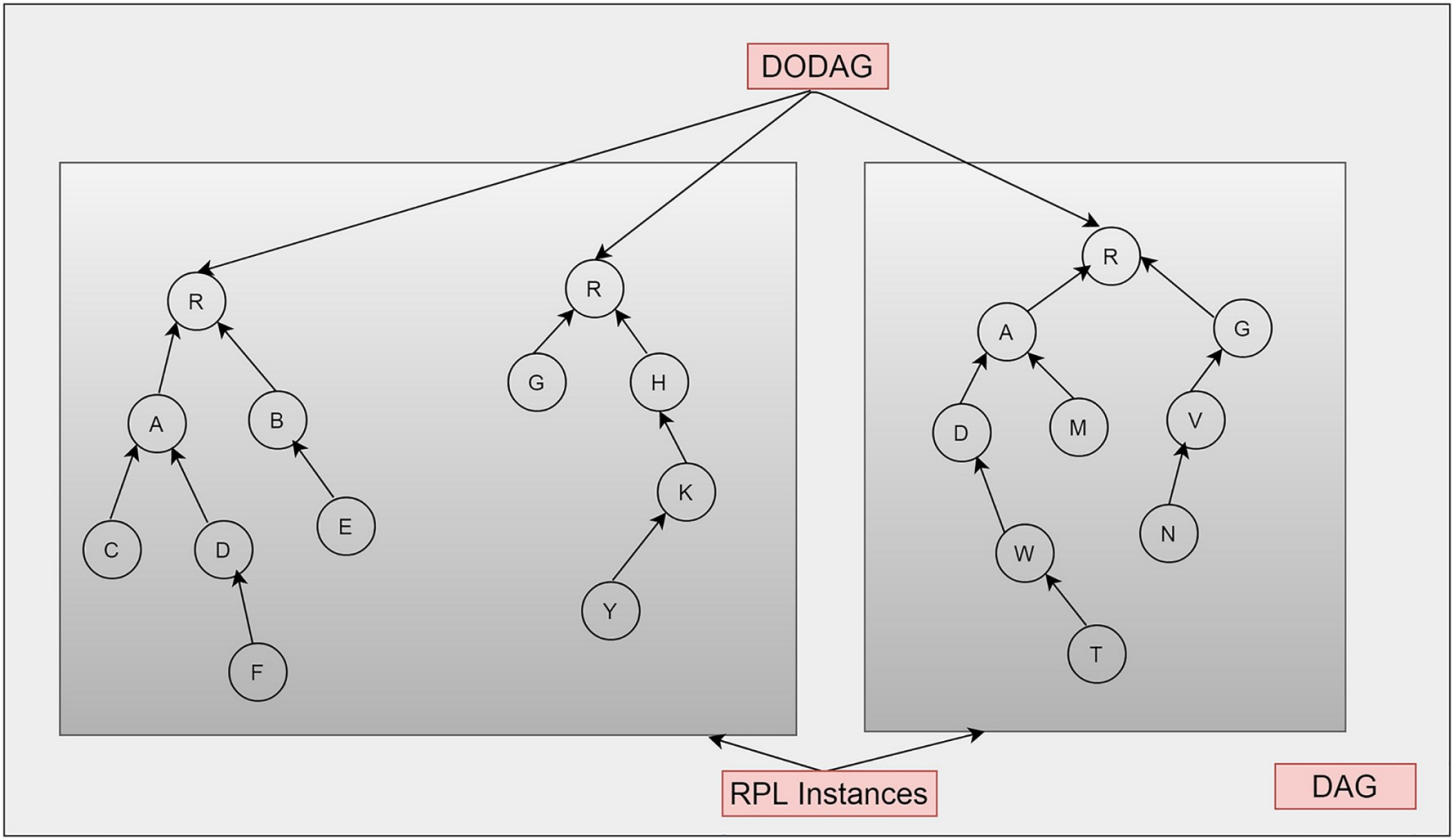
Number of attacks detected.

The experiment evaluated that in the first 10 minutes, all the techniques detected more attacks, as shown in Fig 13. In the next 50 minutes, there is a progressive decrease in attack detection and remains at a constant range of 100-150 in our case, which is still more than the state-of-the-art techniques presented in [42] as SEC-Trust and [8] as DTCM. It can be seen that in the last 5 minutes, the detection rate of THC-RPL started to increase. At the same time, it kept declining for both the state-of-the-art techniques. Sooner both of the techniques (i.e., [8, 42]) may fail to detect a substantial number of attacks. In our case, the improvement in the detection of attacks is due to the registration of every node at the Root node or BR and having a list of mobile nodes’ identities at every child node. Suppose any node creates a new Sybil identity or steals the identity of the neighbor node. In that case, our technique depends on the matrices energy and forwarding behavior and node identity identification by child node. It instantly informs the sink node for quick identification of Sybil nodes. While in [8, 42], the node takes different matrices and decisions. In our technique, overall trust is computed for the node by considering the direct and indirect trust using the last observation-only and the dual identity identification, which helps better detect Sybil attacks.

### 6.2 Packet loss ratio

**Fig 14:**
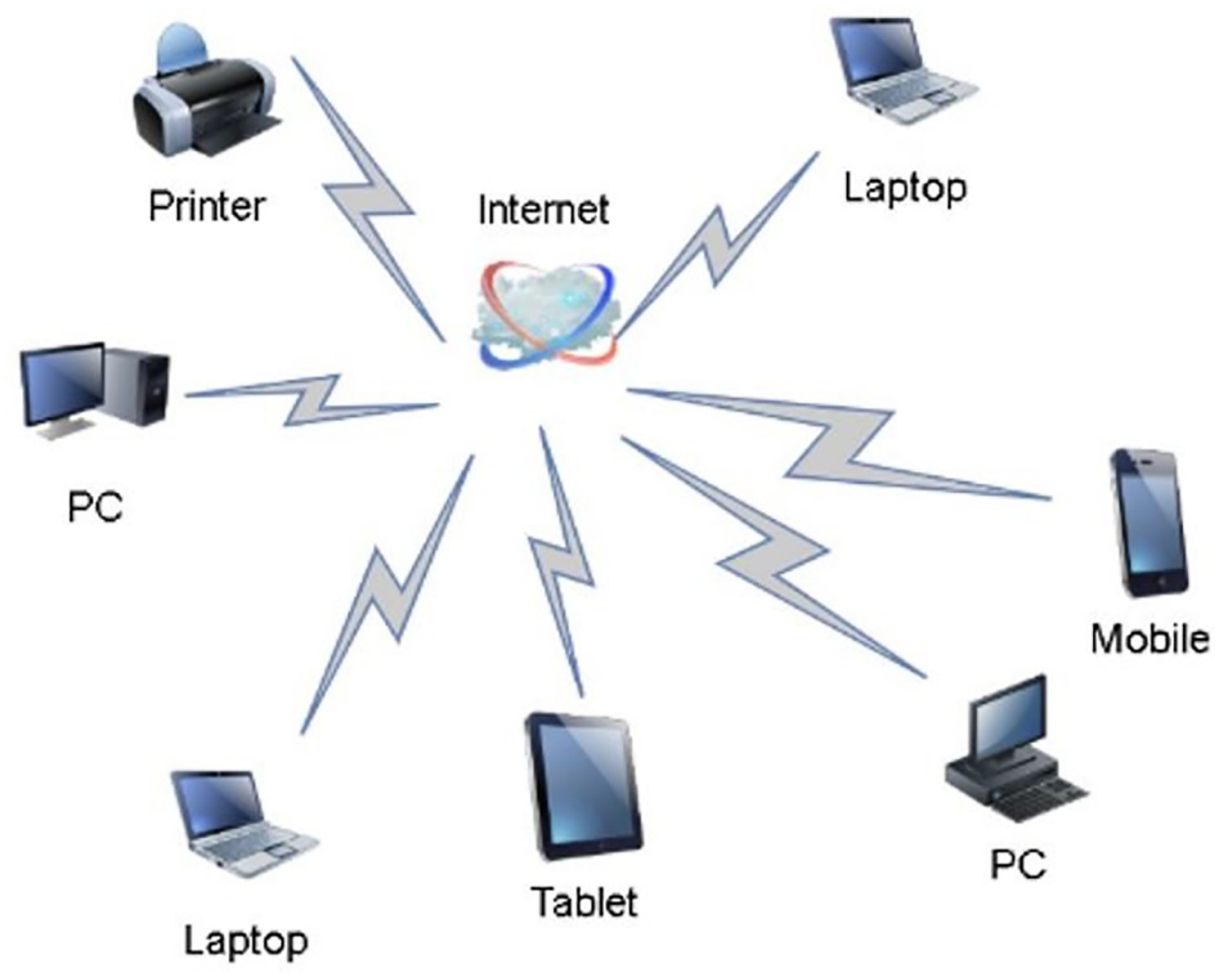
Percentage of packet loss ratio.

It is evident from the simulation in Fig 14 that SEC-Trust has a high packet loss ratio, the nodes ID 6 to onward have an 80% to 90% packet loss ratio. SEC-trust did not have any mechanism to handle the attacks in a mobile scenario. While in DCTM, the packet loss pattern lies in the range of 30-40%. In the DCTM technique, the running node takes the decision independently using different matrices, maintaining the topology while detecting and isolating the attack, which results in a high packet loss ratio. While our technique, due to the detection and isolation of malicious nodes by root nodes, results in a low packet loss rate. The packet loss ratio remains in the ratio of 15-25% only in our technique.

### 6.3 Average energy consumption of nodes

**Fig 15:**
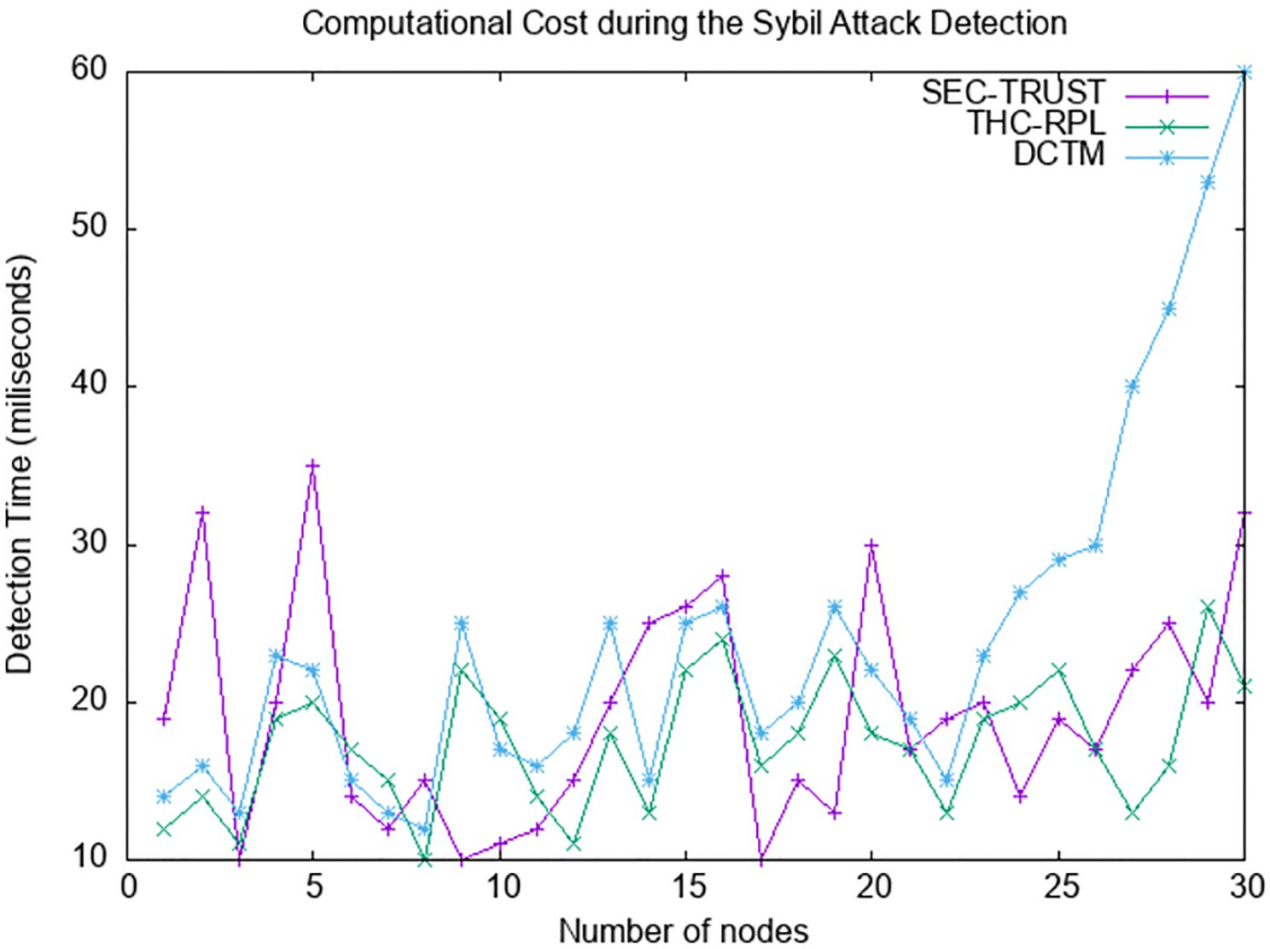
Energy consumption of nodes.

It is clear from the simulation results, shown in Fig 15 that the energy consumption at the node-level is way greater on SEC-trust and DCTM compared to the proposed THC-RPL. It is also worth noting that THC-RPL started conserving more energy at the node level over time, which is 30% to 40% more than DCTM and SEC-Trust, respectively. In contrast, the energy consumption is remarkably high for the SEC-trust and relatively lesser for DCTM (yet still higher than the THC-RPL). Since they did all trust-related computations at the node level, they badly drained the nodes’ energy. While in THC-RPL, the trust is computed partially at the node level and remaining at the root node that substantially conserved the nodes’ energy. Hence, it indicates clearly that our technique works better than the two and consumes less energy.

### 6.4 Energy consumption of network

**Fig 16:**
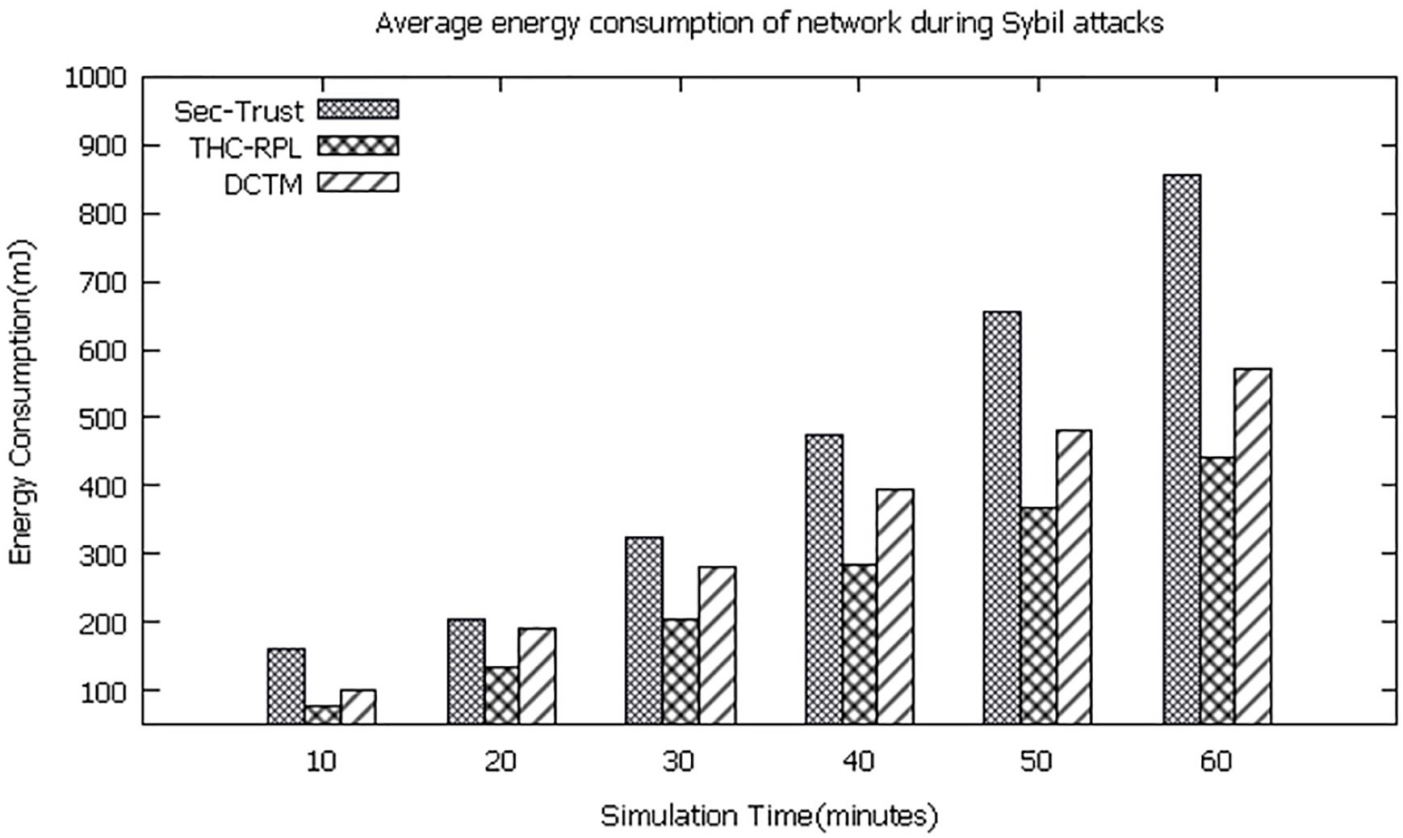
Energy consumption of network.

These experimentation results show that the average energy consumption in the network is better for the proposed technique than the rest of the two techniques. It is clear from the observation that the network’s energy consumption is greater in SEC-Trust than in DCTM and our technique because Sec Trust does not have any mechanism to detect and isolate the mobile node’s new identities and stolen identities. Similarly, DCTM has a mechanism to detect and isolate the Sybil attack’s new identities and stolen identities. However, the computation of the trust model at the node level and reconstruction of DAG of RPL repeatedly results in more energy consumption. While in our technique, the child node and root node collaboratively compute the trust model due to which it consumes less energy that, results in increasing the life span of the network as shown in Fig 16. Overall 50% of the network life span increased in THC-RPL compared to SEC-Trust and DCTM, which does not handle the mobile Sybil attacks efficiently, resulting in more energy consumption in exchange of messages and RPL DAG reconstruction.

### 6.5 Computation cost

**Fig 17:**
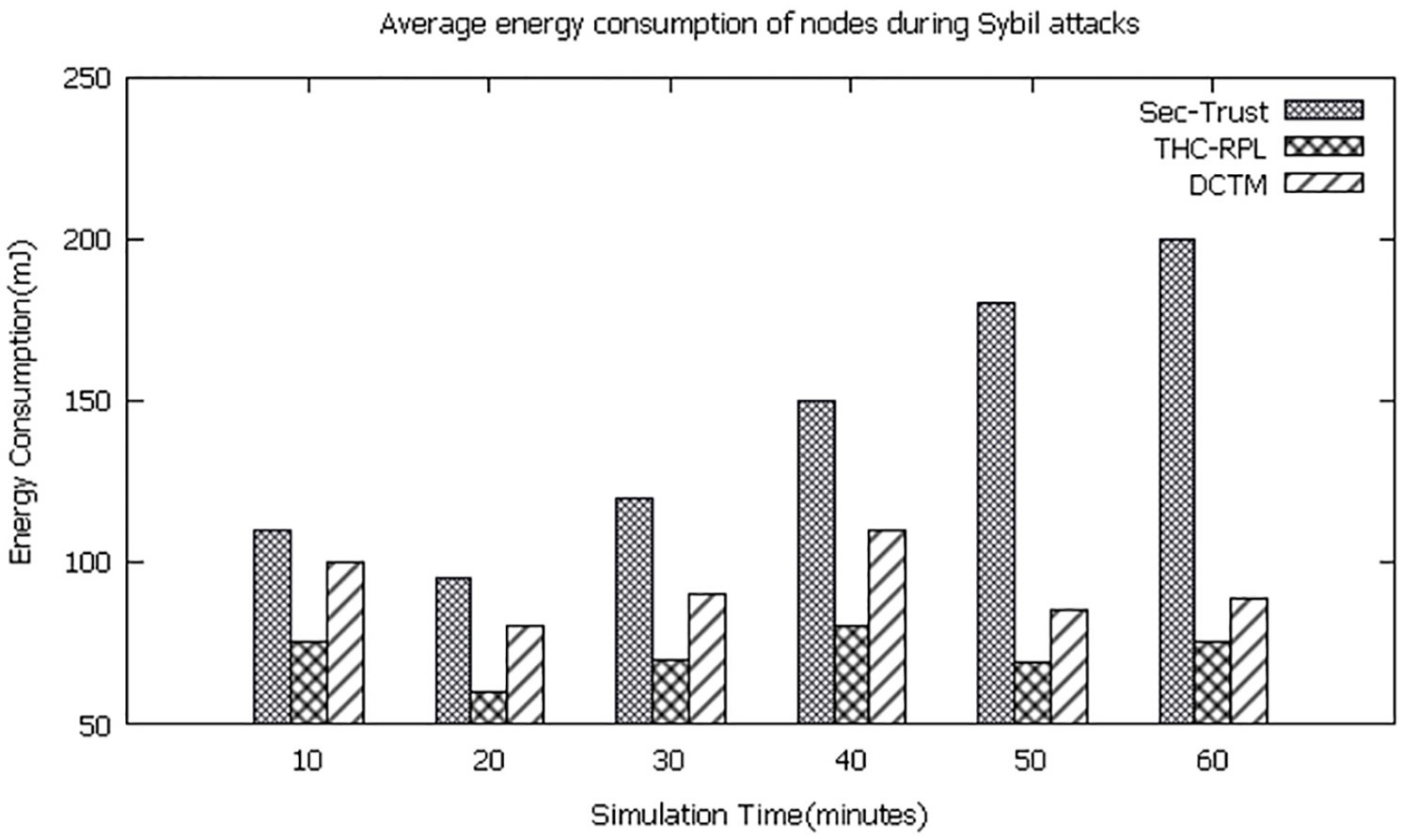
Computation cost.

After network configuration and topology formation, the nodes start regular communication (i.e., sending and receiving data packets). To evaluate the trust of each node, nodes also start exchanging messages (i.e., trust parameters) after topology formation. In this regard, we also compared computation costs among the proposed THC-RPL and the state-of-the-art. The computation cost, in our case, can be defined as the time it takes from exchanging trust parameters to detecting a malicious node. Fig 17 represents the comparison for computation cost during detecting Sybil attacks. It can be seen in the graph that the detection time for all three schemes kept on changing; however, this change is persistent in our case. The DCTM detection time rose exponentially when the number of nodes increased, whereas SEC-trust and the proposed one remained persistent; however, SEC-trust took more time than the proposed one. Due to the presence of the mobile node list to the child nodes, they can easily communicate with each other in our case. Whereas, in the case of static nodes, child nodes decide the direct trust based on the two metrics, standard transmissions and energy consumption. In the case of mobile node scenarios, the mobile node can check the mobile node list when it starts communicating with the other nodes. They start communication and calculate the direct trust if it occurs in the network. In THC-RPL, all computations do not occur at the node level as child nodes do not take the decision to declare the node is malicious and cut off the communication with that node. In THC-RPL, the root node takes the final decision. Once the node is declared malicious, the RPL DAG is reconstructed, resulting in secure communication. In THC-RPL computations done at the root node, the detection time increases 2% to 3%; however, it does not affect the overall network performance, as shown in Fig 17.

### 6.6 Communication cost

**Fig 18:**
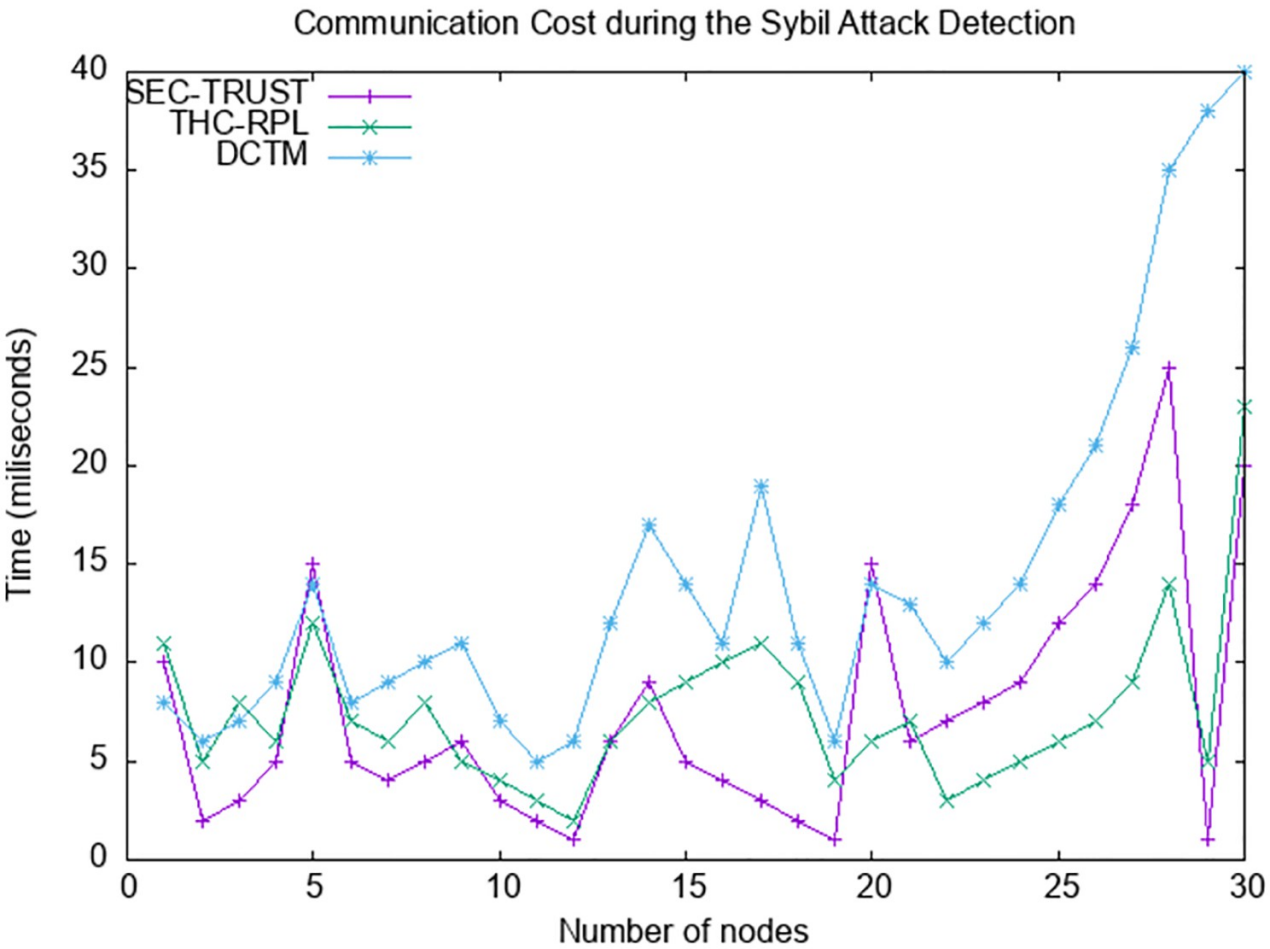
Communication Cost.

Fig 18 depicts the communication cost comparison among SEC-trust, DCTM, and the proposed THC-RPL. The communication cost in our case is more stable and less than that of SEC-trust and DCTM. It is also shown that the DCTM communication cost exponentially exceeds the increase in nodes’ numbers. It is since the SEC-trust and DCTM deal with the static and mobile nodes’ topology after observing the behavior of the nodes. They evaluate trust immediately after topology formation, increasing message exchange. For example, they break the link with the Sybil node and reconstruct the topology. Reconstructing results in the formation of the topology of RPL, which poses substantial communication overhead. While in the case of the proposed THC-RPL, the nodes check the node’s behavior first, and if it lies below the trust level, it will inform the Root node instead of taking an abrupt decision to break the link and reconstruct the DAG. The root node decides whether a node is malicious or not and starts the process of reconstruction if needed. Therefore, the communication cost in THC- RPL is less compared to the SEC-trust and DCTM. DCTM and SEC-trust compute the trust at the node level; all nodes decide to declare the node as a malicious node and reconstruct the RPL DAG. In THC-RPL, as the computations are done at root nodes and the final decision to declare the node as Sybil, the reconstruction is initiated only by a single source. The proposed model handles the communication cost by periodically updating the direct trust value to the root node. This communication does not affect the network performance because it works periodically, unlike the rest of the state-of-the-art not immediately informing the root node when the trust value does not meet the threshold value. That is why inn THC-RPL, there is 10% less exchange of messages than the rest of the state-of-the-art methodologies.

### 6.7 Storage cost

**Fig 19:**
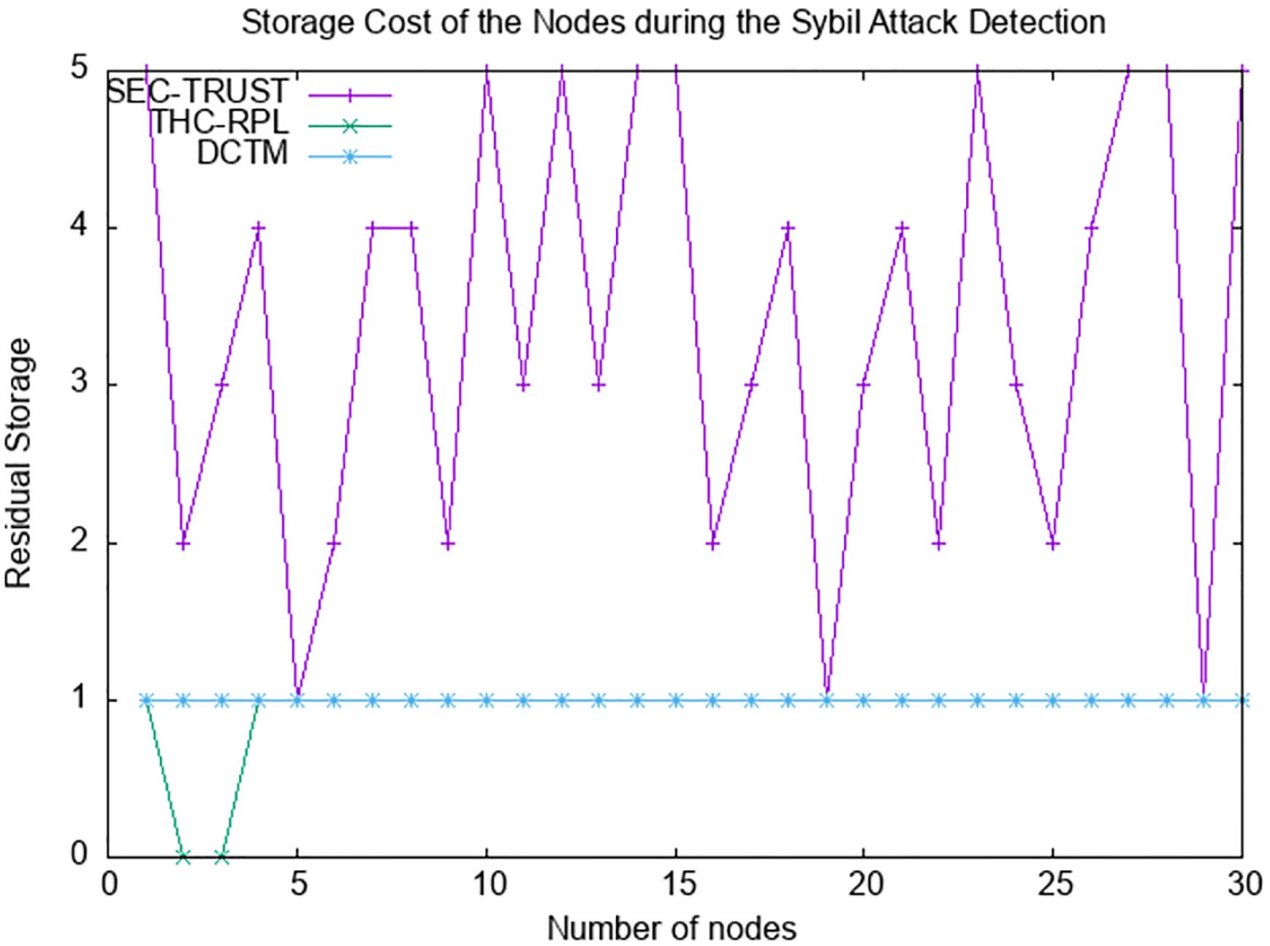
Storage cost.

Tmote Sky sensor motes feature Texas Instrument MSP430 microcontroller. It is an ultra-low power microcontroller featuring 10 KB of RAM and 48 KB of flash. In THC-RPL, only a few bits are stored, as depicted in Fig 19. Whereas in SEC-trust and DCTM, nodes observe the behavior of their neighboring nodes and keep a list at the node level. The decisions about malicious and benign nodes are also made and saved there, which increases storage utilization at the node level. However, in the case of THC-RPL, the nodes only have a list of the mobile nodes; therefore, only a few bits of storage are occupied. It is to be noted that keeping the list at the node level does not affect the performance of the network. The information about the nodes requires only 2-5 bits. Therefore, in contrast to SEC-trust and DCTM, the proposed THC-RPL does not require more space.

## 7. Conclusions and future work

This paper introduced the Trust-Based Hybrid Cooperative (THC-RPL) protocol. In THC-RPL, every child node observes their directly connected neighbor node’s behavior, calculates the node’s DT, and transmits this observation to the Root node. The root node calculates the trust value of the DT and IDT. Based on the opinion of different neighbors, the node’s trust value is calculated, which tells whether the node is malicious or not. The root node identifies the dual identity of a single node (i.e., a Sybil identity). This study introduced a system in which every node is registered with the root node. The root node created two types of lists: malicious and trusted or legitimate node lists. The global trust value was calculated at the root node that helped form a malicious node list. This malicious node list was propagated through the Root node to all child nodes of the network. In this way, malicious nodes were isolated from the network, and only the trusted nodes could participate in routing. The proposed work was evaluated through network performance metrics, such as several attacks detected, packet loss ratio, and the average energy consumption of nodes. In all evaluation metrics, our methodology performs better than the state-of-the-art, and it ultimately increased the life span of the IoT network. In the future, we aim to evaluate the THC-RPL in a real testbed configuration.
